# A CLDN18.2‐Targeted Nanoplatform Manipulates Magnetic Hyperthermia Spatiotemporally for Synergistic Immunotherapy in Gastric Cancer

**DOI:** 10.1002/advs.202413913

**Published:** 2025-02-28

**Authors:** Xueying Wang, Hui Hui, Jing Han, Ting Guo, Yiding Wang, Lin Meng, Cong Chen, Jie He, Xiaoyong Guo, Fuyu Zhong, Hong Du, Jie Tian, Xiaofang Xing, Yang Du, Jiafu Ji

**Affiliations:** ^1^ Key Laboratory of Carcinogenesis and Translational Research (Ministry of Education) Gastrointestinal Cancer Translational Research Peking University Cancer Hospital & Institute Beijing 100142 China; ^2^ CAS Key Laboratory of Molecular Imaging Institute of Automation Chinese Academy of Sciences Beijing 100190 China; ^3^ University of Chinese Academy of Sciences Beijing 100080 China; ^4^ Key Laboratory of Carcinogenesis and Translational Research (Ministry of Education) Department of Biochemistry and Molecular Biology Peking University Cancer Hospital & Institute Beijing 100142 China; ^5^ School of Engineering Medicine & School of Biological Science and Medical Engineering Beihang University Beijing 100191 China; ^6^ Key Laboratory of Big Data‐Based Precision Medicine (Beihang University) Ministry of Industry and Information Technology of China Beijing 100191 China; ^7^ State Key Laboratory of Holistic Integrative Management of Gastrointestinal Cancers Beijing Key Laboratory of Carcinogenesis and Translational Research Gastrointestinal Cancer Centre Peking University Cancer Hospital & Institute Beijing 100142 China

**Keywords:** CLDN18.2, gastric cancer, immunotherapy, magnetic hyperthermia, molecular imaging, nanoparticle

## Abstract

Precision treatment of gastric cancer requires specific biomarkers, and CLDN18.2 emerges as a promising target for patients’ stratification and therapeutic guidance. In 563 cases, 54.4% of patients are identified as CLDN18.2‐positive, with CLDN18.2 expression negatively correlated with immune‐related factors like PD‐L1, indicating a “cold” tumor microenvironment. Here, a novel CLDN18.2 monoclonal antibody 1D5 is created with superior high specificity and affinity, and the antibody‐dependent fluorescence‐magnetic nanoparticle is developed for specific detection and magnetic hyperthermia (MHT). Under the assistance of sensitive fluorescence and deep‐penetrating magnetic particle imaging for tracing and timing the optimal nanoparticle dosage, MHT induces robust immunogenic response via DNA mismatch repair and tumor‐associated antigen release. It recruits CD11c^+^ dendritic cells, compensates PD‐1 in CD8^+^ T cells, and enhances CD86^+^ macrophage polarization. The combination of anti‐PD‐1 therapy increased TNF‐α and IFN‐γ secretion and further boosted the cytotoxic efficacy of CD8^+^ T cells. Excellent therapeutic efficacy is found simultaneously on cell‐derived allografts and patient‐derived xenografts based on this spatiotemporally manipulated strategy, presenting a therapeutic option for enhancing responsiveness to immunotherapy for CLDN18.2‐positive individuals.

## Graphical abstract

CLDN18.2 is a highly selective biomarker exposed in gastric cancer. A CLDN18.2‐specific antibody 1D5 conjugated fluorescence‐magnetic nanoparticle is developed for image‐guided precision theragnostics. Under the assistance of sensitive FMI and deep‐penetrating MPI for tracing and timing the optimal nanoparticle dosage, combining α‐PD1 therapy with nanoparticle‐mediated MHT can potentiate the treatment effectiveness against gastric cancer.

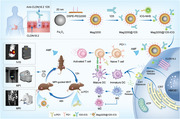

## 1 Introduction

Gastric cancer (GC) remains the fifth most prevalent malignancy and the third leading cause of cancer‐related deaths worldwide.^[^ ^1^, ^2^, ^3^ ^]^ Considering its vague initial signs, 70–90% of patients have progressed to advanced stages at initial diagnosis, leading to an unfavorable overall prognosis.^[^ ^4^ ^]^ Immune checkpoint blockade (ICB) against programmed cell death 1 (PD‐1) and its ligand programmed cell death ligand 1 (PD‐L1) presents an opportunity to treat advanced GC. Unfortunately, the high heterogeneity and large population of PD‐L1‐negative GC limit the number of prospective ICB responders, leading to unsatisfactory immune efficacy.^[^ ^5^ ^]^ Therefore, there is an unmet need for more effective therapeutic targets and techniques to improve GC treatment.

Claudin‐18 isoform 2 (CLDN18.2) is a highly selective biomarker typically expressed in normal gastric mucosal epithelial cells.^[^ ^6^, ^7^, ^8^, ^9^, ^10^, ^11^ ^]^ During malignant transformation, the disruption of cell polarity in the gastric and gastro‐oesophageal junctions may lead to increased exposure to CLDN18.2, rendering it more accessible to therapeutic antibodies.^[^ ^8^, ^9^, ^10^, ^11^, ^12^ ^]^ Recent studies indicated that GCs exhibiting positive CLDN18.2 expression (moderate‐to‐strong CLDN18.2 expression in ≥ 40% tumor cells) constitute ≈49–85% of GCs.^[^ ^8^, ^13^, ^14^ ^]^ Compared to commonly encountered molecular markers such as the human epidermal growth factor 2 (HER2), overexpressed in 15–25% of gastric/gastroesophageal junction (G/GEJ) cancers;^[^ ^15^, ^16^, ^17^, ^18^ ^]^ epidermal growth factor receptor (EGFR), amplified or overexpressed in 5–10% patients;^[^ ^19^, ^20^ ^]^ and PD‐L1, overexpressed in 14–69% patients with combined positive score ≥ 1–50 used for targeted and immunotherapeutic interventions,^[^ ^13^ ^]^ CLDN18.2 exhibits significantly elevated expression levels and improved tissue specificity. Furthermore, CLDN18.2 therapeutic antibody IMAB362 has demonstrated remarkable efficacy in CLDN18.2‐positive and HER2‐negative GCs.^[^ ^21^, ^22^ ^]^ Enhancing the affinity and specificity of CLDN18.2 monoclonal antibodies is critical for accurately differentiating between various CLDN18 subtypes within the complex tumor microenvironment. Such improvements could significantly augment therapeutic efficacy and minimize drug‐related side effects.

Recent studies have reported that ^89^Zr and ^124^I labeled CLDN18.2 antibodies could function as non‐invasive tracers for GC diagnosis through positron emission tomography functional imaging.^[^ ^23^, ^24^, ^25^ ^]^ These studies pave the way for nanomedicine‐based therapies specifically targeting CLDN18.2 and exploring synergistic immunotherapeutic strategies. Magnetic particle imaging (MPI) as an emerging imaging modality has gained considerable attention owing to its extremely high sensitivity and unlimited image depth.^[^ ^26^, ^27^, ^28^ ^]^ When combined with magnetic hyperthermia (MHT), MPI signals are traced directly from the magnetic nanoparticle (MNP) magnetic moment in the presence of a magnetic solid gradient,^[^ ^29^ ^]^ and the specific absorption rate (SAR) dose is predicted to ensure the MHT safety and efficacy.^[^ ^30^ ^]^ Intriguingly, MNPs modified with biological tumor‐targeting peptides improve the delivery uniformity of MHT in solid tumors, indicating a significant potential for navigating MNP‐mediated MHT.^[^ ^31^, ^32^, ^33^ ^]^ This capability supports the in vivo tracing of MNP pharmacokinetic processes such as absorption, distribution, metabolism, and excretion. Consequently, it provides valuable guidance to physicians in selecting the optimal time points for subsequent MHT.

Mild MHT with temperature controlled ≈42–45 °C significantly modulates the tumor immune microenvironment (TIME).^[^ ^34^, ^35^ ^]^ Exposure to an alternating magnetic field (AMF) causes MNPs to heat cancer cells and release tumor‐associated antigens (TAAs). This process promotes dendritic cells (DCs) maturation and activates CD8^+^ T cells and natural killer cells.^[^ ^35^, ^36^, ^37^, ^38^ ^]^ ICB therapy enhances cytotoxic T‐cell levels, preventing tumor immune evasion, inhibiting distant tumor growth, and establishing long‐term immune memory. Furthermore, cytotoxic T cells release cytokines, activating other immune cells and sustaining durable antitumor responses.

In this study, the clinicopathological characteristics of CLDN18.2 were comprehensively analyzed in the TCGA database and a 563 GC cohort. We successfully developed a novel CLDN18.2‐specific monoclonal antibody 1D5 with 6 fold and 10 fold higher affinity than that of IMAB362 and IMAB294 (commercially used), respectively. Then, we utilized it as a targeting element to develop a CLDN18.2‐targeted fluorescent superparamagnetic nanoplatform (SPIO@1D5‐ICG) that could facilitate precise, safe, and efficient MHT under the guidance of MPI. The theragnostic function of SPIO@1D5‐ICG was investigated across cell lines, cell‐derived allografts (CDA), and patient‐derived xenografts (PDX). We found that MPI‐guided SPIO@1D5‐ICG, in combination with MHT, could effectively treat GC by efficiently inducing immunogenic cell death (ICD) in tumor cells. Hence, combining anti‐PD1 (α‐PD1) therapy with SPIO@1D5‐ICG mediated MHT can further potentiate the treatment effectiveness against GC cancer. It provides a promising therapeutic option for CLDN18.2‐positive patients, improving their response to immunotherapy.

## 2 Results and Discussion

### 2.1 CLDN18.2 is Prevalently Expressed in GCs and Correlated with Poor Early Prognosis and Immunosuppressive Status

**Figure 1:**
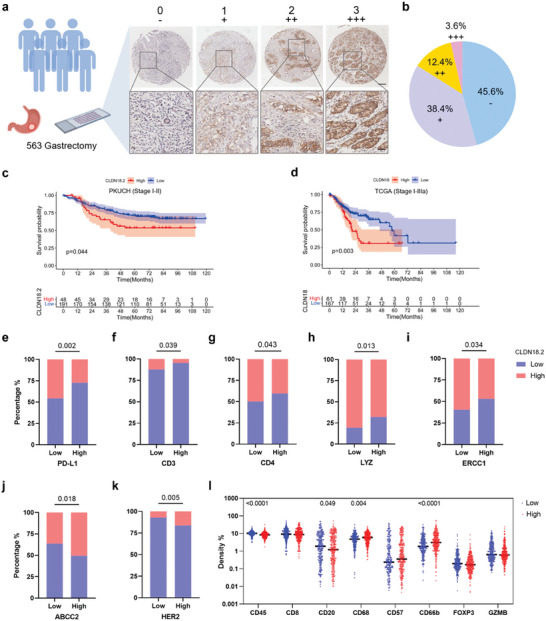
Clinical characteristics between low and high expression of CLDN18.2 in GC. a) CLDN18.2 protein expression was assessed by immunohistochemistry in PKUCH gastric tissue microarray samples. n = 563. Scale bar: 200 µm; scale bar in magnified pictures: 50 µm. b) Score percentage of CLDN18.2 expression analyzed from (a). c,d) Overall survival of GC patients with high versus low expression of CLDN18.2 in stages I‐II of PKUCH IHC data (c) and CLDN18 in stages I‐IIIa of TCGA database (d) for GCs. Statistical significances were calculated via a log‐rank test. e–k) Correlation between CLDN18.2 expression and other cancer signaling molecules and immune features. Statistical significances were calculated via the Cochran–Mantel–Haenszel 2 test. LYZ: Lysozyme; ERCC1: Excision repair cross‐complementation group 1; ABCC2: ATP‐binding cassette sub‐family C member 2. l) Comparison of immune cell infiltration of GCs in PKUCH cohort with low or high CLDN18.2. Statistical significances were calculated via the Wilcoxon test.

Confirming that CLDN18.2 is a potential target in GC, the mRNA expression of *CLDN18* was first analyzed in the Cancer Genome Atlas (TCGA) database. Results revealed elevated CLDN18 expression levels in both GCs and normal gastric tissues, with no discernible differences (Figure S1, Supporting Information). We examined the CLDN18.2 protein expression in tissue microarray samples from the PKUCH cohort containing 563 GC cases with distinctive pathological characteristics (Table S1, Supporting Information). The CLDN18.2 expression levels ranged from 0 (negative) to 3+ (strong), with levels above one defined as CLDN18.2 positive (high expression) (**Figure** **1**a). CLDN18.2‐positive GCs accounted for ≈54.4% of our tested cohort, consistent with previous reports (Figure 1b; Figure S2, Supporting Information).^[^ ^39^, ^40^ ^]^ CLDN18.2 expression levels were higher in intestinal‐type, well‐differentiated, and early‐stage GCs (Table S1, Supporting Information).

In early‐stage GCs (I‐IIIa stages in TCGA database and I‐II stages in PKUCH database), positive CLDN18.2 expression was correlated with shorter OS (Figure 1c,d). However, in all stages, patients with higher CLDN18.2 did not show a significantly worse prognosis than those with low levels (Figure S3a, b, Supporting Information). High PD‐L1 level has been recognized as a favorable prognostic marker in several studies and has been validated in TCGA and PKUCH databases (Figure S3c,d, Supporting Information).^[^ ^13^, ^41^ ^]^ Subgroup analysis revealed the protective role of PD‐L1, specifically within the CLDN18.2‐negative cohort (Figure S3e, f, Supporting Information). Interestingly, within the CLDN18.2‐positive subgroup, PD‐L1 ceased to exert a prognostic impact, indicating a potential correlation between CLDN18.2 and PD‐L1 (Figure S3g,h, Supporting Information). The results of the univariate and multivariate COX analyses for OS were shown in Figures S4 and S5 (Supporting Information) with subgroups according to clinicopathological parameters.

The correlation between CLDN18.2 and other specific molecules was analyzed. Notably, CLDN18.2 displayed negative correlations with PD‐L1 (Figure 1e), CD3 (Figure 1f), CD4 (Figure 1g), lysozyme (Figure 1h), and excision repair cross‐complementation group 1 (Figure 1i), and positive correlations with ATP‐binding cassette sub‐family C member 2 (Figure 1j) and HER2 (Figure 1k). It also revealed a lower proportion of CD45^+^ immune cells and CD20^+^ B cells and a higher proportion of CD68^+^ macrophages and CD66b^+^ neutrophils in the CLDN18.2‐positive group (Figure 1l). These findings were consistent with several studies,^[^ ^42^, ^43^, ^44^ ^]^ suggesting that the CLDN18.2‐positive group displayed a relatively suppressed tumor immune microenvironment, and a synergistic therapeutic approach targeting CLDN18.2 and ICB may yield enhanced efficacy.

### 2.2 Generation of Monoclonal Antibody 1D5 with Higher Binding Affinity to CLDN18.2

**Figure 2:**
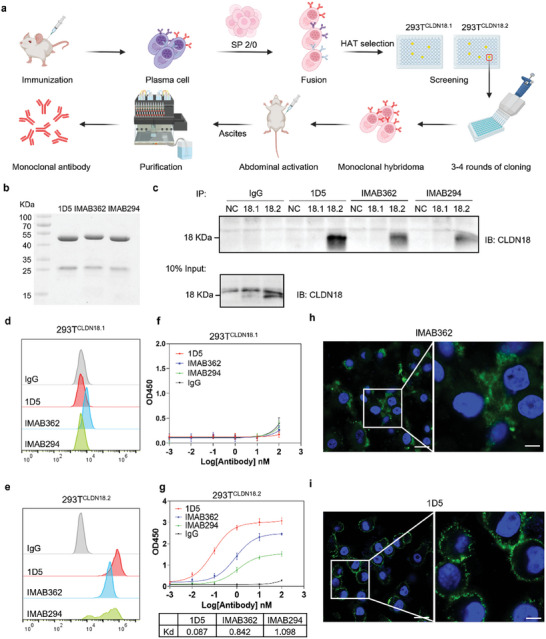
Generation and characterization of CLDN18.2 monoclonal antibody 1D5. a) Overview of murine monoclonal antibody generation and purification. SP2/0: murine myeloma cell line; HAT selection: Hypoxanthine‐aminopterin‐thymidine selection. b) Coomassie blue staining of purified anti‐CLDN18.2 antibodies 1D5, IMAB362 and IMAB294. c) Immunoprecipitation analysis of IgG, 1D5, IMAB362 and IMAB294 anti‐CLDN18.2 antibodies. NC: 293T cell lysis; 18.1: 293T^CLDN18.1^ cell lysis; 18.2: 293T^CLDN18.2^ cell lysis. d, e) Flow cytometry histogram of 293T, 293T^CLDN18.1^, and 293T^CLDN18.2^ cells incubated with IgG, 1D5, IMAB362, or IMAB294 antibodies. f,g) Binding affinity measurements of 1D5, IMAB362, IMAB294 and IgG to 293T^CLDN18.1^ and 293T^CLDN18.2^ cells. Kd: dissociation constant. h,i) Immunofluorescence staining of living MFC^CLDN18.2^ cells with IMAB362 and 1D5. Scale bar: 20 µm; scale bar in magnified pictures: 5 µm.

Optimizing the binding affinity and specificity of CLDN18.2 monoclonal antibodies remains a critical focus. We successfully generated a series of monoclonal antibodies with high activity and selectivity following the strategy of mouse‐derived CLDN18.2 hybridoma production and purification in **Figure** **2**a. The candidate antibody 1D5 (Figure S6, Supporting Information) was identified through pharmacological, pharmacokinetic, and safety evaluations, and it was subsequently subjected to a head‐to‐head comparison with the commercially available antibodies IMAB362 and IMAB294 from Ganymed Pharmaceuticals. Coomassie blue staining confirmed that 1D5, IMAB362, and IMAB294 all possess high purity and concentration (Figure 2b). Immunoprecipitation (IP) assays compared the specificity of the antibodies 1D5, IMAB362, IMAB294, and IgG for binding CLDN18.2. As shown in Figure 2c, 293T^CLDN18.2^ but not 293T^CLDN18.1^ was successfully immunoprecipitated by all three antibodies, which proved the binding specificity to CLDN18.2. Furthermore, 1D5 exhibited a superior affinity for CLDN18.2 compared to the commercial antibodies. Flow cytometry analysis further corroborated the enhanced specificity and affinity of 1D5 for CLDN18.2, as demonstrated by the size of the overlapping region in fluorescence spectra and the positioning of characteristic peaks (Figure 2d,e). ELISA results also confirmed that 1D5, IMAB362, and IMAB294 all specifically bound to CLDN18.2, with 1D5 showing over 1000 fold selectivity for CLDN18.2 over CLDN18.1, whether in free protein or cell surface protein forms (Figure 2f,g). Notably, the affinity of 1D5 for CLDN18.2 was found to be 6 fold and 10 fold than that of IMAB362 and IMAB294, respectively (1.22 nM compared to 6.95 and 12.5 nM). Immunofluorescence analysis of MFC^CLDN18.2^ cells incubated with 1D5 and IMAB362 further indicated that 1D5 displayed stronger membrane binding specificity (Figure 2h,i). These findings suggested that the 1D5 antibody exhibited superior affinity and specificity for CLDN18.2 compared to commercial antibodies IMAB362 and IMAB294, highlighting its enhanced potential for clinical translation.

### 2.3 Synthesis and Characterization of SPIO@1D5‐ICG Nanoparticle with Good Imaging, Targeted Binding, and Biocompatibility Properties

**Figure 3:**
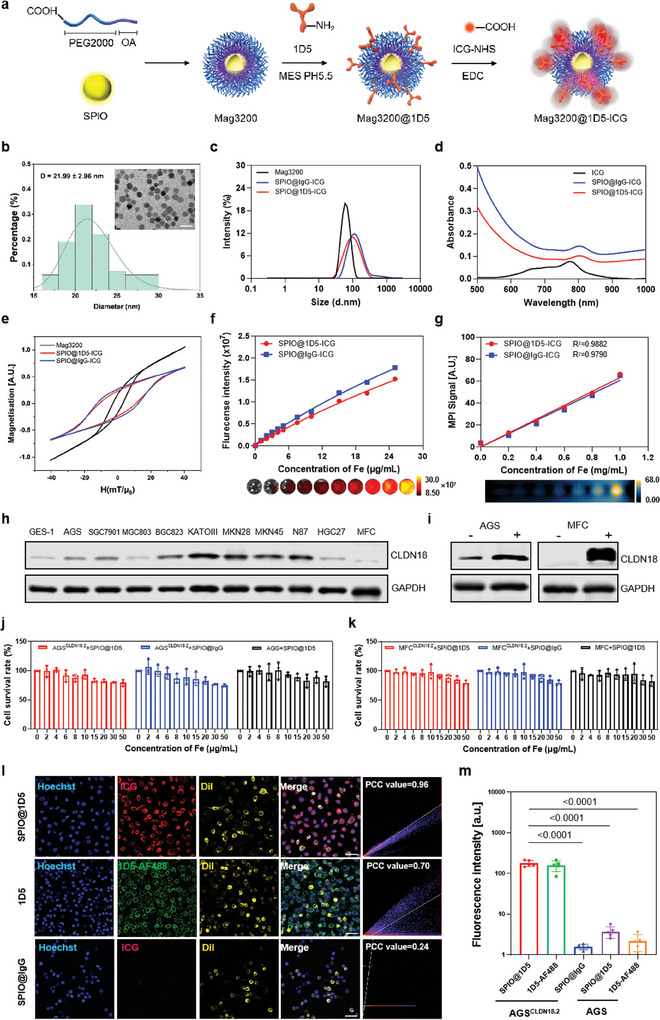
Physicochemical properties and biocompatibility of SPIO@1D5‐ICG in vitro. a) Schematic demonstration of preparing SPIO@1D5‐ICG. Magnetic nanoparticles modified with carboxyl‐terminated DSPE‐PEG2000 were conjugated to the monoclonal antibody 1D5 via an amide reaction, followed by a fluorescence reaction with ICG‐NHS. b) Representative transmission electron microscopy (TEM) images of SPIO (inset: size distribution of SPIO analyses). Scale bars = 50 nm. c) UV–vis absorbance spectra of ICG, SPIO@1D5‐ICG, and SPIO@IgG‐ICG. d) Dynamic light scattering (DLS) analysis of SPIO@1D5‐ICG, SPIO@IgG‐ICG and SPIO is shown as number‐weighted % frequency. e) Magnetic particle spectrum measurements for Mag3200, SPIO@1D5‐ICG, and SPIO@IgG‐ICG. f) Fluorescence intensity of SPIO@1D5‐ICG increased with rising Fe concentrations using the IVIS spectrum. g) Magnetic particle imaging (MPI) signals of SPIO@1D5‐ICG nanoparticles increased with rising Fe concentrations. h) CLDN18.2 protein expression in GC and gastric cell lines determined by western blot (WB). i) WB images of CLDN18.2 overexpression in MFC and AGS cells. j,k) In vitro cytotoxicity of MFC^CLDN18.2^ and AGS^CLDN18.2^ cells incubated with increasing concentrations of SPIO@1D5‐ICG. l) CLSM images of AGS^CLDN18.2^ cells incubated with SPIO@1D5‐ICG or SPIO@IgG‐ICG. Internalized nanoparticles (red) and Cell membrane staining (Dil, yellow) colocalization. Pearson's correlation coefficient (PCC) was calculated using the analyze‐colocalization‐color 2 plugin in Image J. Scale bar: 50 µm. m) Quantitative analysis of fluorescence intensity in SPIO@1D5‐ICG, 1D5‐AF488 and SPIO@IgG‐ICG incubated AGS^CLDN18.2^ cells, as well as in SPIO@1D5‐ICG and 1D5‐AF488 treated AGS cells. All data were presented as mean ± SD. One‐way ANOVA and Dunnett calculated statistical significance multiple comparisons test (j,k,m).

Magnetic nanoparticles modified with carboxyl‐terminated DSPE‐PEG2000 (Mag3200) were first conjugated with the amino groups of CLDN18.2 monoclonal antibody 1D5 in 1‐ethyl‐3‐(3‐dimethylaminopropyl) carbodiimide (EDC) cross‐linking agent. The indocyanine green N‐hydroxysuccinimide ester (ICG‐NHS) dye was then labeled to the amino groups of 1D5, resulting in the final Mag3200@1D5‐ICG product (denoted below SPIO@1D5‐ICG or SPIO@1D5), for the imaging and accurate magnetic heating of GC. Synthesis procedures are shown in **Figure** **3**a. The antibody conjugation rate calculated for SPIO@1D5‐ICG was 26.01% (Figure S7a, Supporting Information), with a fluorophore‐to‐protein (F/P) ratio of 3.71 (Figure S7b,c, Supporting Information).

The conjugation, morphology, surface zeta potential, magnetothermal properties, and crystal form of SPIO@1D5‐ICG were characterized using Fourier transform infrared (FTIR) spectroscopy, transmission electron microscopy (TEM), dynamic light scattering, magnetic particle spectroscopy, X‐ray diffraction (XRD) and a fiber thermocouple (Figure S7d,e, Supporting Information), indicating the stable physicochemical properties of the nanomedicine. The FTIR analysis confirmed the successful conjugation of 1D5 and ICG to the SPIO surface, as evidenced by the appearance of characteristic peaks (Figure S7d, Supporting Information). TEM observed good monodispersity of SPIO in Mag3200 (Figure 3b) and SPIO@1D5‐ICG (Figure S7e, Supporting Information), with average sizes at 21.99 ± 2.96 nm and 20.81 ± 2.46 nm, respectively. The SPIO@1D5‐ICG hydrodynamic size (Figure 3c) was ≈92.89 nm, which increased by 33.82 nm to Mag3200 (59.07 nm). The zeta potential (Figure S7f, Supporting Information) of the SPIO@1D5‐ICG changed from −28.06 to −20.82 mV after camouflaging. The absorbance spectrum exhibited a broad and elevated absorption between 780 and 850 nm (Figure 3d). The magnetization curves showed that Mag3200 magnetization was ≈1.7 times higher than that of SPIO@1D5‐ICG and SPIO@IgG‐ICG (Figure 3e). This result also revealed that SPIO@1D5‐ICG and SPIO@IgG‐ICG produced similar coercive forces via hysteresis losses under AMF, significantly improving the energy‐dissipation rate through potent magnetic‐to‐heat induction. Magnetothermal heating/cooling curves showed that Mag3200, SPIO@1D5‐ICG, and SPIO@IgG‐ICG increased to above 50 °C under AMF, a widely used treatment temperature. Furthermore, the magnetothermal stability was evaluated, indicating similar reversible magnetothermal properties for the three nanoparticles (Figure S7g, Supporting Information). The XRD peaks are consistent with the crystal planes of Fe_3_O_4_. All peaks were indexed to Fe_3_O_4_ (JCPDS 19–0629, Figure S7h, Supporting Information). To assess the fluorescence molecular imaging (FMI) and MPI properties, we analyzed the FMI and MPI signals at different Fe concentrations and found that the fluorescence intensity positively correlated with an increased SPIO@1D5‐ICG concentration (Figure 3f). The MPI linear regression results and corresponding images showed that the MPI signals increased as samples increased (Figure 3g). Characterization analysis showed that SPIO@1D5‐ICG was successfully synthesized and retained its FMI and MPI properties.

CLDN18.2 expression was further screened in human and murine gastric cancer cell lines and normal human gastric tissue. Results showed low CLDN18.2 expression levels in AGS and MFC wild‐type cell lines (Figure 3h). Lentiviral vectors harboring human and murine CLDN18.2 plasmids were individually transfected into AGS and MFC cells (Figure S8, Supporting Information). Upregulated CLDN18.2 expression in both AGS and MFC cells was detected by western blotting (Figure 3i; Figures S9 and S10a,b, Supporting Information), and the surface increased CLDN18.2 levels were also verified by flow cytometry (Figure S10c–f, Supporting Information). The viability of either CLDN18.2 overexpressed or control cell lines changed slightly after incubation with gradually increasing concentrations of the nanoparticle (0–50 µg mL^−1^ of Fe), indicating its good biocompatibility and permissible toxicity (Figure 3j,k). Confocal laser scanning microscopy (CLSM) images displayed stronger signals from the ICG channel in SPIO@1D5‐ICG‐treated AGS^CLDN18.2^ (Figure 3l; Figure S11a, Supporting Information) and MFC^CLDN18.2^ (Figure S12a, Supporting Information) cells. The localization of CLDN18.2 was seen at the cell membranes of AGS^CLDN18.2^ and MFC^CLDN18.2^ cells; the less binding signal was found in AGS and MFC wild‐type cell lines. The colocalization of fluorescence in different groups was presented (Figures S11b and S12b, Supporting Information), and mean fluorescence intensity in two cell types among different groups was statistically significant, illustrating that more nanoparticles were attached and internalized by tumor cells after conjugation with 1D5 (Figure 3m; Figure S12c, Supporting Information). Our results confirmed that SPIO@1D5‐ICG could effectively target CLDN18.2 overexpressing GC cells.

### 2.4 The Tumor Accumulation and Prolonged Blood Circulation of SPIO@1D5 by FMI and MPI Dual‐Modality Imaging In Vivo

**Figure 4:**
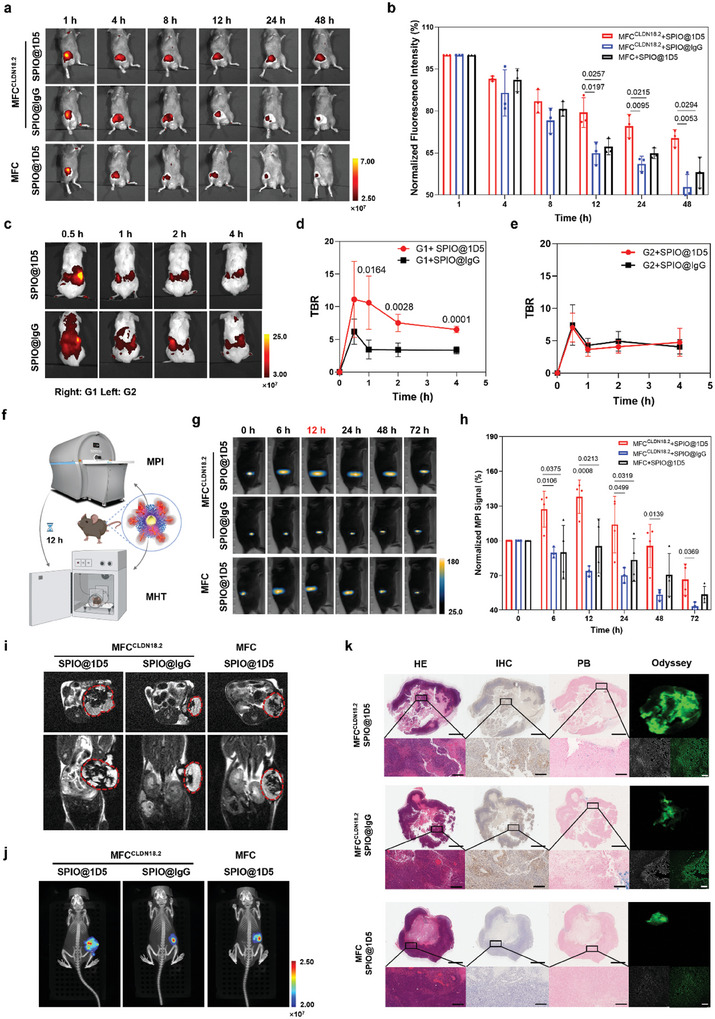
The in vivo biodistribution and tumor targeting of SPIO@1D5 were traced by FMI and MPI dual‐modality imaging. a) Representative fluorescence images of intravenous injection of SPIO@1D5 or SPIO@IgG in MFC^CLDN18.2^ and MFC subcutaneous xenograft tumor models at different time points. b) Quantitative comparison of normalized tumor‐to‐background ratio (TBR) of fluorescence intensity. Data were expressed as means ± SD (n = 3). Skin fluorescence intensity was selected as the background. c) Fluorescence images of PDX models (G1: right; G2: left) after intravenous injection of SPIO@1D5 or SPIO@IgG. d,e) Quantitative comparison of TBR for SPIO@1D5 or SPIO@IgG injected G1 (d) and G2 (e). Data were expressed as means ± SD (n = 4). f) Schematic for MPI tracing and timing the dosage of SPIO@1D5 in MHT. g) MPI images of intratumoral injection of SPIO@1D5 in MFC^CLDN18.2^ reached the maximum at 12 h, compared with SPIO@IgG injected MFC^CLDN18.2^ and SPIO@1D5 injected MFC allografts at different time points. h) Quantitative comparison of normalized MPI signal of the nanoparticles. Data were expressed as means ± SD (n = 4). i) MRI images of allografts after intratumoral injection for 12 h. The red dotted circle represents the location of the tumor. j) FMI‐CT 3D reconstruction of xenografts after intratumoral injection for 12 h. k) Ex vivo multimodality profiling of FFPE tissue sections by H&E, CLDN18.2 IHC, Prussian blue staining and Odyssey CLx Imaging System. Scale bar: 2 mm; scale bar in H&E, IHC, and Prussian blue staining magnified pictures: 200 µm; scale bar in fluorescence images: 100 µm. H&E: hematoxylin and eosin staining; IHC: immunohistochemistry; PB: Prussian blue staining. Statistical significances were calculated via the unpaired two‐tailed Student's *t*‐test (d,e), two‐way ANOVA, and Dunnett multiple comparisons test (b,g).

Sensitive FMI was displayed to examine the biodistribution of SPIO@1D5 in vivo. It produced stronger fluorescent signals in MFC^CLDN18.2^ cells at the tumor site, suggesting the superior specificity and binding activity of SPIO@1D5 in CLDN18.2‐overexpressing tumors. SPIO@IgG also generated weak but detectable signals due to the enhanced permeability and retention effect and rapidly decayed in fluorescence intensity (**Figure** **4**a,b). Ex vivo imaging revealed that the MFC^CLDN18.2^ tumor treated with SPIO@1D5 showed the most vital signals (Figure S13a, b, Supporting Information).

PDXs have become the gold standard for clinical drug development because of their similarity to original tumors dissected from patients. Two cases of GC PDX with different CLDN18.2 expression (G1 and G2) were used and displayed identical clinicopathological characteristics (Figure S14, Supporting Information). SPIO@1D5 and SPIO@IgG were intravenously injected and monitored for 4 h (Figure 4c). Notably, SPIO@1D5 quickly accumulated in the CLDN18.2‐positive PDX group (G1) and remained higher compared with the SPIO@IgG‐treated groups (Figure 4d), while less targeting and no significant difference in fluorescence was observed in the CLDN18.2‐negative PDX (G2, Figure 4e). Ex vivo FMI showed a significantly stronger tumor signal in SPIO@1D5‐treated G1 compared with G2 and SPIO@IgG‐treated G1 (Figure S15a, b, Supporting Information), which was further verified through quantitative analysis (Figure S15c, Supporting Information). These results highlight the excellent specific target binding and biocompatibility of SPIO@1D5 in vivo.

To maintain higher tumor retention and circumvent off‐target hepatic accumulation, SPIO@ID5 was administered intratumorally for in vivo imaging and further guidance of precise MHT (Figure 4f). As an emerging imaging technique, MPI exhibited exceptional sensitivity and image depth in tissues (Figure S16, Supporting Information). MPI imaging (Figure 4g) and quantitative analysis (Figure 4h) showed an increased MPI signal at 6 h‐post injections, reaching a peak at 12 h with homogeneous distribution. After 72 h, the SPIO@1D5 signal decreased by half. On the contrary, SPIO@IgG‐treated MFC^CLDN18.2^ and SPIO@1D5‐treated MFC exhibited no significant nanoparticle diffusion and were metabolized more rapidly. These results suggested that MHT might achieve the optimal therapeutic effect when performed 12 h after nanoparticle administration, guided by MPI imaging. Assessment of MRI (Figure 4i) and fluorescence 3D‐CT (Figure 4j; Figure S17, Supporting Information) after nanoparticle injection for 12 h further validated the biodistribution and metabolic behavior of SPIO@1D5. Ex vivo images (Figure S18a, Supporting Information) and quantitative analysis (Figure S18b, Supporting Information) of the major organs and tumors at 72 h post‐injection demonstrated significantly stronger tumor signals in the SPIO@1D5 treated MFC^CLDN18.2^ group. Prussian blue staining detected less iron accumulation in the liver and spleen, confirming the preferential retention of SPIO@1D5 in tumors (Figure S19a, b, Supporting Information). Pathological sections of tumor tissues at 72 h, including H&E staining, CLDN 18.2 IHC, Prussian blue staining, and Odyssey quad‐modal imaging, corroborated the uniform distribution of SPIO@1D5 within the MFC^CLDN18.2^ tumor at the pathological level (Figure 4k). H&E staining of the mouse heart, liver, spleen, lungs, and kidneys showed no discernible toxic side effects of the SPIO@1D5 (Figure S20, Supporting Information). The exceptional sensitivity and quantification capability of MPI facilitates the real‐time tracing of the metabolism and distribution of magnetic nanoparticles within tumors, thereby assisting clinicians in selecting the optimal time window for magnetic hyperthermia and drug delivery.

### 2.5 SPIO@1D5‐Mediated MHT Combined with α‐PD1 Therapy Presents Strong Antitumor Efficacy in CDA and PDX Models

**Figure 5:**
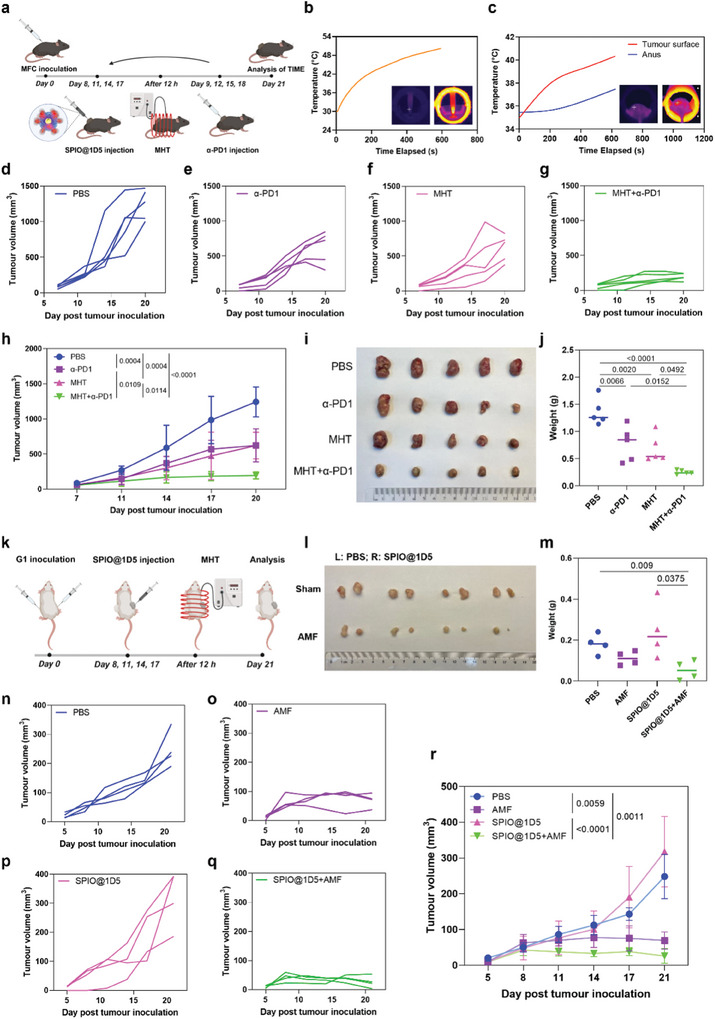
Potent antitumor activity of SPIO@1D5 mediated MHT combined with anti‐PD1 antibody in subcutaneous CLDN18.2 positive GC model. a) Schematic timeline of the therapy process for 615 mice bearing MFC^CLDN18.2^ tumors. b) Temperature−time curves of SPIO@1D5 (1.0 mg mL^−1^) under AMF (20A, 353KHZ, 1.6KW). Infrared imaging of SPIO@1D5 upon AMF treatment for 10 min. c) Temperature−time curves of prior 12 h SPIO@1D5 injected tumor skin and axillary temperature of mice under the same AMF monitored by fiber optic thermometry. d–g) Individual tumor growth curves for all four groups. h) Tumour growth curves in MFC^CLDN18.2^ tumor‐bearing mice after different treatments. i) Photographs of harvested MFC^CLDN18.2^ tumours on day 21 after PBS, α‐PD1, MHT and combined therapies. Data were expressed as means ± SD (n = 5). j) Tumour weights of different groups taken on day 21 of Figure 5i. k) Schematic timeline of the MHT therapy process for the G1 PDX model. l) Photographs of harvested PDX tumors on day 21 after different treatments (n = 4; L: left; R: right). m) PDX tumor weights of different groups taken on day 21 of Figure 5l. n–q) Individual PDX tumor growth curves for all four groups. r) PDX tumor growth curves after different treatments. Data were expressed as means ± SD (n = 4). Statistical significances were calculated via one‐way ANOVA and Tukey multiple comparisons test.

We studied the antitumor efficacy of SPIO@1D5‐mediated MHT under MPI guidance. As shown in **Figure** **5**a, the intratumoral injection of SPIO@1D5 and MPI‐guided MHT was performed in MFC^CLDN18.2^ tumor‐bearing 615 mice and followed by α‐PD1 therapy. Upon AMF treatment, the temperature of an aqueous suspension of SPIO@1D5 significantly increased by as much as 20 °C for 10 min with a SAR of 142.95 ± 10.46 W g^−1^ (Figure 5b; Figure S21a, Supporting Information). For mice injected with SPIO@1D5, the temperature of the tumor surface increased from 34 to 41 °C with a SAR of 45.33 ± 3.49 W g^−1^, while the anus temperature remained stable below 38 °C (Figure 5c; Figure S21b, Supporting Information). These results indicated that SPIO@1D5 exhibited excellent MHT performance and achieved stable thermal effects for antitumor therapy in vivo under AMF. As shown in Figure 5d–h, the tumor volumes in the treatment group (MHT + α‐PD1) were significantly suppressed. In contrast, the PBS‐treated group showed rapid growth, and the α‐PD1‐only and MHT‐only groups partially inhibited tumor growth. These results demonstrated the superior therapeutic effect of the MHT and α‐PD1 combination. After all monitoring studies, the tumors were dissected (Figure 5i) to measure their weights (Figure 5j). The obtained average tumor weight of the MHT + α‐PD1 group diminished to 0.270 ± 0.075 g, which was much lower than that from other groups: control group (1.364 ± 0.310 g), α‐PD1‐only group (0.740 ± 0.445 g) and MHT‐only group (0.688 ± 0.259 g). Compared with PBS treatment, these results revealed that α‐PD1 or MHT partially controls the tumor growth rate, whereas the combination of ICB and MHT demonstrates the most robust antitumor efficacy.

To further explore the impact of SPIO@1D5 and AMF on mouse tumor growth, PDXs with high CLDN18.2 expression were bilaterally implanted into NOD/SCID mice. All PDX models were intratumorally injected with SPIO@1D5 on the right side and PBS on the left side, followed by either AMF or no exposure (Figure 5k). Tumour debulking images (Figure 5l) and weight recordings (Figure 5m) after 21 days revealed the best inhibitory effect observed with the AMF and SPIO@1D5 combination therapy (Figure 5n–r). Intriguingly, significant tumor suppression was also exhibited in SPIO@1D5 untreated sites, attributed to the abscopal effects of immune leakage.^[^ ^45^ ^]^ The routine blood test results (Figure S22, Supporting Information) and blood biochemical indicators (Figure S23, Supporting Information) in the mice treated with PBS, SPIO@1D5, or SPIO@1D5 combined with AMF showed no apparent abnormalities.

### 2.6 The Combined MHT and α‐PD1 Activate TIME by Recruiting DCs and Stimulating Cytotoxic T Cells

**Figure 6:**
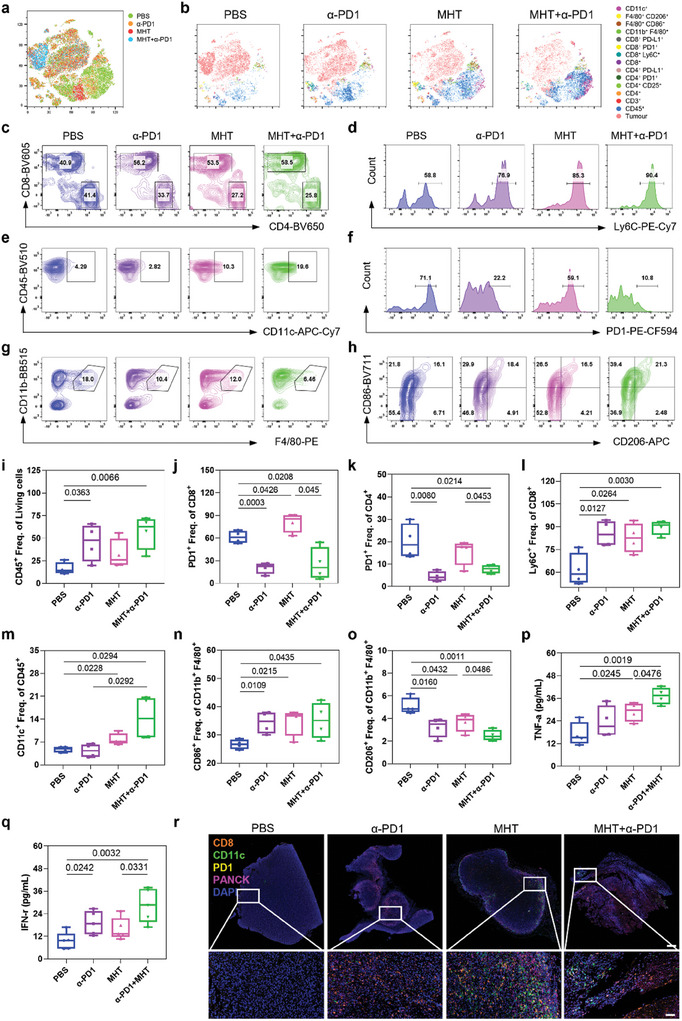
Effect of combined MHT with α‐PD1 on tumor immunological profile in MFC^CLDN18.2^ models. a) T‐distributed stochastic neighbor embedding (t‐SNE) analysis on flow cytometry events of all harvested tumors. b) t‐SNE of all donor groups is used to define and compare single‐cell clusters between different treatments. c‐h) Representative flow cytometry analysis of CD4^+^ or CD8^+^ T cells frequency (c, gated on CD3^+^ T cells), Ly6C histogram profile (d, gated on CD8^+^ T cells), CD11c^+^ DCs frequency (e, gated on CD45^+^ cells), PD1 histogram profile (f, gated on CD8^+^ T cells), CD11b^+^ and F4/80^+^ macrophages frequency (g, gated on CD45^+^ cells), CD86^+^ M1 or CD206^+^ M2 cells frequency (h, gated on CD11b^+^ F4/80^+^ macrophages) inside the tumour with PBS, α‐PD1, MHT and MHT + α‐PD1 therapies. i–o) Quantification of (i) CD45^+^ frequency in living cells, (j) PD1^+^ frequency in CD8^+^ T cells, (k) PD1^+^ frequency in CD4^+^ T cells, (l) Ly6C^+^ frequency in CD8^+^ T cells, (m) CD11c^+^ DCs frequency in CD45^+^ cells, (n) CD86^+^ frequency in CD11b^+^ and F4/80^+^ cells, (o) CD206^+^ frequency in CD11b^+^ and F4/80^+^ cells. p, q) Serum levels of TNF‐α (p), IFN‐γ (q) in the indicated groups. r) Representative multiplex fluorescence microscopy images on FFPE tissue sections showing tumor‐infiltrating immune cells stained with CD11c: green, CD8: Orange, PD1: yellow, PanCK: pink, and DAPI blue. Scale bar: 600 µm; scale bar in magnified pictures: 60 µm. Data were expressed as means ± SD (n = 4). Statistical significances were calculated via one‐way ANOVA and Tukey multiple comparisons test.

Encouraged by the excellent antitumor capacity of the combined hyperthermia‐immunotherapy, we next analyzed whether SPIO@1D5‐augmented MHT therapy could synergistically create an immunogenic “hot” TIME with ICB to boost the antitumor immune response. MFC^CLDN18.2^ tumor‐bearing 615 mice received the same treatment described above: PBS, α‐PD1, MHT, and MHT + α‐PD1. Tumour tissues were isolated on day 21 to examine the TIME components (Figure S24, Supporting Information). The t‐distributed stochastic neighbor embedding (t‐SNE) algorithm (**Figure** **6**a) showed a significant increase in CD11c^+^ DCs clustering in the MHT and combination groups, along with remarkably diminished tumor clustering in the combination group (Figure 6b). For detailed analysis, the amount of CD8^+^ in CD3^+^ T cell groups increased, and the CD4^+^ groups decreased, especially in the MHT + α‐PD1 group (Figure 6c), with an elevated proportion of Ly6C^+^ within CD8^+^ T cells (Figure 6d), indicating higher infiltration and a more active status of immune cells within the tumor. Furthermore, MHT and MHT + α‐PD1 treatments remarkably increased the number of CD11c^+^ DCs (Figure 6e) in tumor tissues, revealing that MHT efficiently promoted intratumoral DCs recruitment. The α‐PD1 group effectively suppressed PD1 expression levels in CD8^+^ T cells (Figure 6f). In the mononuclear macrophage system, the standalone use of α‐PD1 and MHT reduced the number of CD11b^+^ F4/80^+^ macrophages, with a synergistic effect observed upon their combination (Figure 6g). α‐PD1 and MHT facilitated macrophage polarisation toward CD86^+^ M1 while inhibiting CD206^+^ M2 polarisation and combination therapy enhanced this effect (Figure 6h).

The data showed that the overall proportion of immune cells (CD45^+^) increased in the α‐PD1 and combination groups (Figure 6i), with noticeable upregulation of CD8^+^ T cells (Figure S25a, Supporting Information), and the number of CD4^+^ T cells decreased (Figure S25b, Supporting Information) in the MHT group. The total number of T cells (CD3^+^, Figure S25c, Supporting Information) and Tregs (CD4^+^ and CD25^+^, Figure S25d, Supporting Information) showed no significant changes. Intriguingly, the MHT treatment upregulated the PD1 expression in CD8+ T cells (Figure 6j). It might reverse the depleted PD‐1 state in CD8+ T cells, thus enhancing the efficacy of ICB treatment (Figure 6j,k). Ly6C^+^ in CD8^+^ T cells significantly increased in the three treatment groups, suggesting increased CD8^+^ T cell activity (Figure 6l). The number of CD11c^+^ DCs rose substantially in the MHT and combined treatment groups (Figure 6m), indicating that MHT recruited more DCs. The number of CD11b^+^ monocytes also increased (Figure S25e, Supporting Information), and the number of macrophages significantly decreased (Figure S25f, Supporting Information) in the combination group. The number of CD86^+^ M1 macrophages increased (Figure 6n), whereas that of CD206^+^ M2 macrophages significantly decreased (Figure 6o), indicating that MHT combined with ICB promoted macrophage polarisation from M0 to M1, while impeded M0 to M2 polarisation. In summary, MHT reversed immunosuppressive cold tumors into immune‐activating hot tumors, and combined with anti‐PD1 treatment, significantly increased the antitumor response. Serum ELISA detected a significant increase of tumor necrosis factor‐α (TNF‐α) and interferon‐gamma (IFN‐γ) in the combination group (Figure 6p,q), and the interleukin‐6 (IL‐6) levels increased exclusively in the MHT group (Figure S25h, Supporting Information).

To further validate the activation status of the TIME from a spatial perspective, representative cases from each group were subjected to multiplex immunofluorescence staining (Figure 6r). The three treatment groups observed an increase in CD8+ T cells, with significant infiltration of CD11c^+^ DCs from the tumor edge toward the center in the MHT and combination groups. Due to its robust antitumor effect, extensive necrotic tissue was observed in the centre of the MHT + α‐PD1 treated tumors. These results indicated that MHT combined with α‐PD1 could activate TIME via recruiting dendritic cells, stimulating cytotoxic T cells, and promoting M1 macrophage polarization.

### 2.7 MHT Induced Apoptosis Via DNA Mismatch Repair, Releasing TAAs that Facilitated DCs Maturation

**Figure 7:**
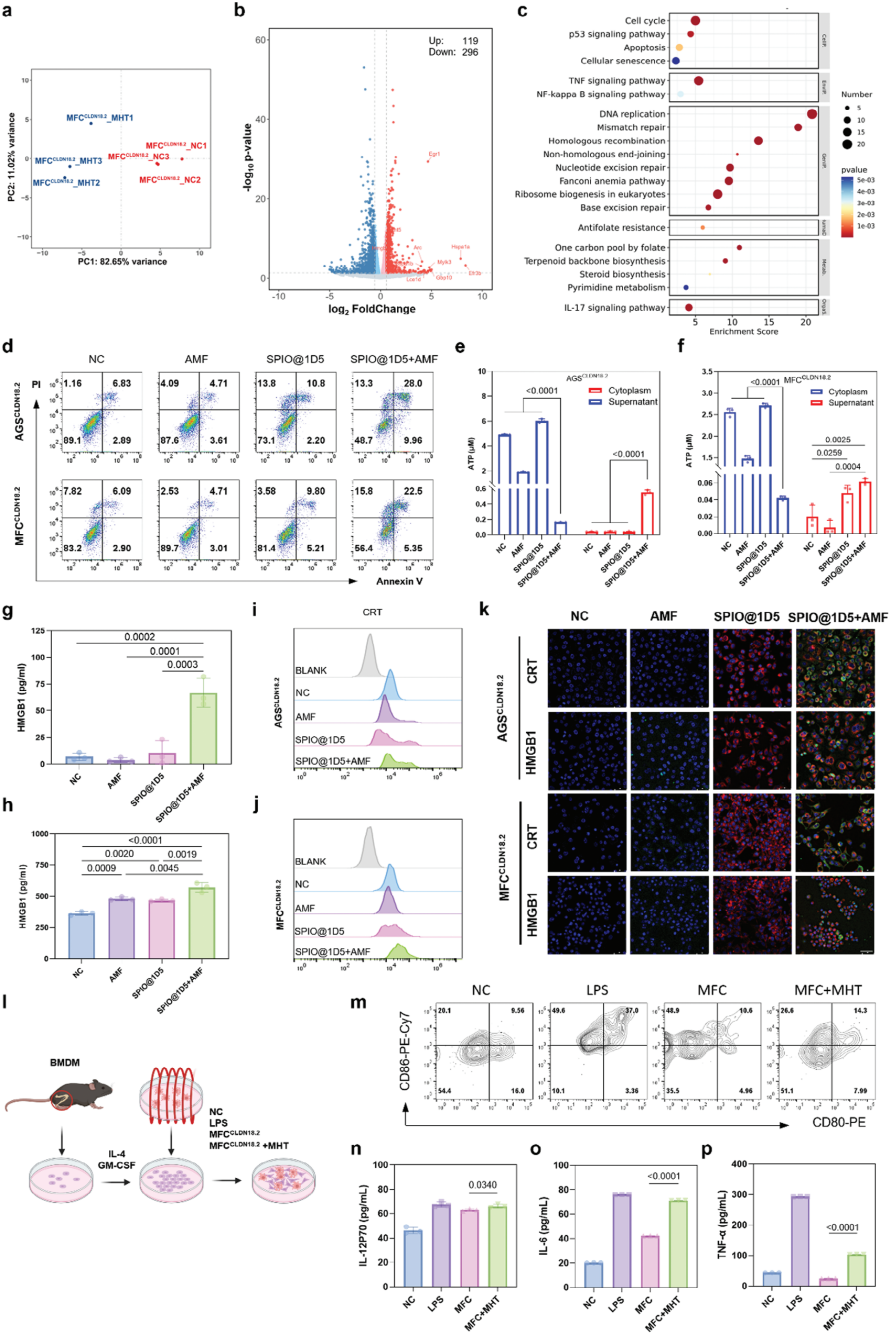
SPIO@1D5‐ICG mediated MHT induces ICD effect and DCs maturation. a) Principal component analysis (PCA) of MFC^CLDN18.2^ cells with or without SPIO@1D5‐ICG mediated MHT (n = 3). b) Volcano plots of MHT‐treated MFC^CLDN18.2^ cells compared with the control group. c) Top 20 enriched KEGG pathways from MHT‐treated MFC^CLDN18.2^ cells compared to the control. d) Flow cytometry analysis of cell apoptosis based on Annexin V‐FITC/PI‐PE staining of MFC^CLDN18.2^ cells with or without MHT. NC: negative control. e‐h) Detection of adenosine triphosphate (ATP) in MFC^CLDN18.2^ (e) and AGS^CLDN18.2^ (f) cytoplasm and supernatants of luciferin‐based ATP assay kit. Flow cytometric examination of calreticulin (CRT) exposure on the surface of MFC^CLDN18.2^ (g) and AGS^CLDN18.2^ (h) cells. i,j) Detection of high mobility group protein B1 (HMGB1) released in the cellular medium of MFC^CLDN18.2^ (i) and AGS^CLDN18.2^ cells (j) by ELISA kit. k) Immunofluorescence of MFC^CLDN18.2^ and AGS^CLDN18.2^ cells stained with CRT‐AF488 and HMGB1‐AF488 antibodies. Scale bar: 50 µm. l) Schematic illustration of BMDCs maturation subjected to NC, LPS, MFC, and MHT‐processed MFC treatment. NC: negative control; LPS: lipopolysaccharide. m) Representative flow cytometry analysis of the population of BMDCs maturation (CD11c^+^ CD80^+^ CD86^+^) after various treatments. n‐p) Quantification of the secretion levels of (n) IL‐6, (o) TNF‐α, and (p) IL12p70 by ELISA kit after different treatments. Data were expressed as means ± SD (n = 3). Statistical significances were calculated via two‐way ANOVA and Tukey multiple comparisons test (e,f), one‐way ANOVA and Tukey multiple comparisons test (g,h), and the unpaired two‐tailed Student's *t*‐test (n‐p).

TIME analysis has revealed that MHT primarily exerts its antitumor immune effects by recruiting and promoting the maturation of DCs. To further explore the specific mechanism of SPIO@1D5 MHT in activating DCs, we simulated the MHT in vitro. MFC^CLDN18.2^ and AGS^CLDN18.2^ cell lines were pre‐treated with 50 µg mL^−1^ SPIO@1D5 for 12 h, followed by AMF treatment under the same conditions for 10 min. Transcriptome sequencing was performed for both MHT‐treated and untreated cells (**Figure** **7**a), and differentially expressed genes were primarily enriched in DNA damage pathways, such as DNA replication, Mismatch repair, and Homologous recombination (Figure 7b,c). Studies have demonstrated that hyperthermia‐induced breaks in both single‐stranded and double‐stranded DNA phosphorylated the C‐terminal serine residues of histone H2AX and ataxia–telangiectasia–mutated protein and suppressed the activities of DNA polymerases and topoisomerases. Meanwhile, hyperthermia amplified the generation of reactive oxygen species (ROS), halted the cell cycle, and impeded DNA replication, culminating in cellular death.^[^ ^46^ ^]^

We compared the apoptotic status of cells in four groups: control, AMF only, SPIO@1D5 only, and SPIO@1D5 with AMF (Figure 7d; Figure S26, Supporting Information). Apoptosis significantly increased when the nanoparticles and AMF were present simultaneously. Consistently, the decreased cytoplasmic and increased secreted adenosine triphosphate (ATP) (Figure 7e,f), the more significant release of high mobility group box 1 (HMGB1) (Figure 7g,k,h; Figure S27c, Supporting Information), and the more robust exposure of calreticulin (CRT) (Figure 7i–k; Figure S27d, Supporting Information), were observed in MFC^CLDN18.2^ and AGS^CLDN18.2^ cells treated with SPIO@1D5 + AMF. These results suggested that SPIO@1D5 + AMF released TAAs and induced substantial ICD effects in tumor cells.^[^ ^47^ ^]^

The maturation of bone‐marrow‐derived dendritic cells (BMDCs) was further analyzed to validate the ICD effect induced by SPIO@1D5‐mediated MHT. As schematically illustrated in Figure 7l, BMDCs were isolated from the tibia and femur of 615 seven‐week‐old mice according to a previous study.^[^ ^48^ ^]^ More robust expression of the costimulatory molecules CD80 and CD86 (Figure 7m; Figure S28, Supporting Information), indicators of DCs maturation, was observed in BMDCs co‐incubated with MHT‐treated MFC^CLDN18.2^ cells, demonstrating that MHT‐triggered ICD efficiently produced DCs maturation, as well as a ligand of toll‐like receptor 4 lipopolysaccharide (LPS), a positive control to induce DC maturation by binding with toll‐like receptors (TLRs) on DCs.^[^ ^49^, ^50^ ^]^ Herein, BMDCs stimulated by MHT‐treated MFC^CLDN18.2^ fragments trigger the adequate secretion of immune cytokines, including IL12p70, IL‐6, and TNF‐α (Figure 7n–p). Morphological alterations in BMDCs after treatment with MFC^CLDN18.2^ fragments were similar to LPS (Figure S29, Supporting Information).

## 3 Conclusion

Gastric cancer (GC) is one of the most prevalent malignancies worldwide and is the leading cause of tumor‐related mortality. Common molecular markers in clinical oncology show relatively low expression rates in GC, posing a challenge in pinpointing ideal targets for personalized therapy. This study focused on the tight junction protein CLDN18.2, which is predominantly expressed in most G/GEJ adenocarcinomas and manifests potential for precise detection and treatment.^[^ ^6^ ^]^ Through the examination of CLDN18.2 expression, prognosis, and clinical features in GC within cohorts from both PKUCH and TCGA, we confirmed 54.4% CLDN18.2‐positive GCs, associated with poor prognosis in early stages, negatively correlated with PD‐L1, CD3 and CD4, and closely linked to suppressed TIME. A novel CLDN18.2 monoclonal antibody 1D5‐dependent fluorescence‐magnetic nanoparticle (SPIO@1D5‐ICG) was synthesized and well‐performed from cell lines to CDA and PDX with excellent specific target binding and biocompatibility. Our study found that combining MPI‐guided SPIO@1D5‐ICG with MHT effectively induced ICD in tumor cells, leading to significant eradication of GC. This approach also enhanced the efficacy of ICB therapy by recruiting DCs and restoring PD‐1 in CD8^+^ T cells. It provides a promising therapeutic option for CLDN18.2‐positive patients, improving their response to immunotherapy.

Therapeutic agents targeting CLDN18.2 continue to emerge. Currently, the CLDN18.2 monoclonal antibody IMAB362 has received FDA approval for application as a first‐line therapy in patients diagnosed with locally advanced unresectable or metastatic HER2‐negative GC/GEJ who exhibit CLDN18.2 positivity.^[^ ^21^ ^]^ Moreover, Zhu and co‐workers reported the CLDN18.2‐targeted nuclear imaging,^[^ ^25^ ^]^ indicating the feasibility of utilizing CLDN18.2 for GC diagnosis. Enhancing the affinity and specificity of CLDN18.2 monoclonal antibodies is critical for accurately differentiating between various CLDN18 subtypes within the complex tumor microenvironment. Therefore, we first generated a novel CLDN18.2 monoclonal antibody 1D5 with 6‐fold and 10‐fold affinity higher than IMAB362 and IMAB294, respectively. Then, we designed and successfully synthesized SPIO@1D5‐ICG using a novel CLDN18.2‐specific antibody and conducted comprehensive preclinical evaluations such as pharmacokinetics, toxicity, targeting, and in vivo pharmacodynamics, confirming the feasibility and safety of these nanomedicines. Precise biodistribution and quantitative assessment of nanoparticles within the tumor were achieved using both FMI and MPI modalities. Under the MPI guidance, the nanoparticles demonstrated real‐time visualization of diffusion and distribution within the tumor without depth limitations or toxic side effects, assisting physicians in determining the optimal time window for MHT dosage and AMF application, which further enhanced the precision and thermal efficiency of the nanoparticles in MHT.

Several studies have indicated that T cells are activated upon recognition of tumor antigens, leading to the upregulation of PD‐1 through antigen‐driven T cell receptor signaling.^[^ ^51^, ^52^, ^53^, ^54^ ^]^ Our study proposes a potential regulatory mechanism for SPIO@1D5 in the immune system, particularly in recruiting and promoting DCs maturation. The internalization of SPIO@1D5 by tumor cells released heat upon exposure to AMF, which could induce cell apoptosis via DNA damage and thereby release TAAs that facilitated DCs maturation. Initially, CRT translocated from the endoplasmic reticulum to the cell membrane and interacted with CD91 on antigen‐presenting cells (APCs). Subsequently, HMGB1 translocated from the nucleus to the extracellular space. The release of ATP into the extracellular milieu facilitated the recruitment of APCs to apoptotic tumor sites, thereby enhancing the phagocytosis and clearance of apoptotic tumor cells. These “eat me” signals contributed to the recruitment and maturation of DCs, resulting in the upregulation of PD‐1 on CD8^+^ T cells via antigen presentation. The combination of α‐PD1 therapy significantly amplifies the cytotoxic effect of CD8^+^ T cells, increasing the secretion of TNF‐α, IFN‐γ, and IL‐6 cytokines. Furthermore, the synergy between MHT and ICB therapy fostered macrophage polarisation from M0 to M1 while impeding M0 to M2 polarisation. In summary, the hyperthermia induced by magnetic particles, in combination with ICB therapy, facilitates the activation of tumor immunity, thereby converting “cold” tumors into “hot.”

In conclusion, this study confirmed the pivotal role of CLDN18.2 in GC, characterized by elevated expression levels, early association with unfavorable prognosis, and potential impact on the TIME. A novel CLDN18.2 targeting nanoparticle was developed for image‐guided MHT, which significantly enhanced the efficacy of ICB treatment in CLDN18.2 positive subjects. It provides a novel therapeutic option for individuals with high CLDN18.2 expression but low immunotherapeutic response rates. Concurrently, this study validated the high expression of CLDN18.2 in a large clinical cohort, emphasizing its crucial value in early tumor prognosis assessment and its interaction with immune‐related molecules, such as PD‐L1, CD3, and CD4. It has important implications for early cancer screening, precision treatment, and lymphadenectomy in surgical navigation and exhibits extensive potential for clinical translation.

## 4 Experimental Section

### Ethics and Samples

All associated clinical information was approved for research applications by the Institutional Ethics Committee of Peking University Cancer Hospital and Institute (2021KT15). All animal experiments were performed under the permission of the Animal Experimentation Ethics Committee of Peking University Cancer Hospital (EAEC 2020‐03). The retrospective analysis involved samples from 563 patients who underwent curative gastrectomy for stomach or esophagogastric junction adenocarcinoma at PKUCH. The inclusion criteria were a primary diagnosis of gastric adenocarcinoma, absence of metastasis at diagnosis, and availability of formalin‐fixed paraffin‐embedded tissue with follow‐up data. Two pathologists independently confirmed the presence of tumor cells and assigned tumor, node, and metastasis (TNM) stages. Clinical and follow‐up data were extracted from the hospital databases. The study received institutional review board approval, and written informed consent was obtained from all participants.

### Immunohistochemistry and Evaluation

IHC assays were performed according to established protocols.^[^ ^55^ ^]^ GC and non‐cancerous stomach tissues were stained with primary antibodies at 4 °C overnight, followed by a 30 min incubation with horseradish peroxidase‐conjugated secondary antibody at room temperature. DAB staining was performed, and counterstaining was performed with Mayer's hematoxylin. The samples were stratified based on the intensity and positive area of protein expression by two experienced pathologists into the following categories: intensity < 0.5 and area < 10%, defined as unfavourable (‐); intensity 0.5–1 and area < 80%, intensity 1–2 and area < 50%, intensity 2–3 and area < 10%, defined as weakly positive (+); intensity 0.5–1 and area > 80%, intensity 1–2 and area > 50%, intensity 2–3 and area 10–80%, defined as moderately positive (2+); intensity 2–3 and area > 80%, or intensity = 3, defined as strongly positive (3+).

### Generation of CLDN18.2‐Specific Monoclonal Antibody 1D5

To generate monoclonal antibodies with specificity for CLDN18.2, BALB/c mice were initially immunized with Claudin 18.2 DNA using in vivo jetPEI‐Gal. Subsequent booster immunizations with the same plasmid were administered every three weeks. Three days following the final immunization, spleen cells from one immunized BALB/c mouse were fused with Sp2/0 cells. Supernatants from the resulting hybridoma cultures were screened for anti‐CLDN18.2 antibodies using indirect ELISA. Hybridomas producing specific antibodies were expanded in BALB/c mice to generate ascitic fluid, from which the monoclonal antibodies were purified via protein‐A/G chromatography. The purity and activity of the monoclonal antibodies were assessed by SDS−PAGE, with gels stained using Coomassie Brilliant Blue. Antibody concentrations were determined by measuring absorbance at 280 nm, calculated using the formula C (mg/mL) = OD_280_ × 0.6768.

### SPIO@1D5‐ICG Assembly and Characteristics

Carboxyl‐terminated superparamagnetic iron oxide nanoparticles Mag3200 (20 nm, 2 mg) at a concentration of 1 mg mL^−1^ were mixed with 0.15 M 2‐(N‐morpholino) ethanesulfonic acid (MES) buffer at 37 °C to reach pH 5.5. Subsequently, CLDN18.2 antibody 1D5 and mouse IgG (control), each at a concentration of 0.2 mg mL^−1^, were incubated at 37 °C under stirring conditions for 30 min. Cross‐linking agent EDC (dissolved in 0.015 M MES buffer at pH 5.5) was added and stirred at 37 °C for 3 h, followed by centrifugation at 20 000 g for 30 min. The supernatants were collected for protein quantification. The product was washed and resuspended in distilled water at a final concentration of 1 mg mL^−1^. ICG‐NHS (1 µL from 10 mg mL^−1^ DMSO solution) was added at 37 °C for 1 h. After centrifugation at 20 000 g for 30 min, the supernatant was discarded. Finally, the product was washed and resuspended at a final 1 mg mL^−1^ concentration for subsequent analyses. Mag3200, 1D5, and ICG‐NHS mass ratios were precisely controlled at 10, 0.2–10, and 0.01–0.5.

### Animal Model Studies

Male 615 mice (4–6 weeks of age) were maintained under pathogen‐free conditions in the animal center of PKUCH. MFC cells (4 × 10^5^ in 100 µL) were subcutaneously injected into the right groin of each mouse. The tumor size reached ≈100 mm^3^ before drug administration. SPIO@1D5‐ICG (25 mg kg^−1^) or PBS was injected intravenously or intratumorally, followed by 5 mg kg^−1^ α‐PD‐1 antibody injected intraperitoneally through four injections every three days. Tumour volume was measured using the formula V = L × W^2^ × 0.5 (V, volume; L, length; W, tumor width). PDX GC tissues (2nd passage) were obtained from PKUCH with different CLDN18.2 expression confirmed through IHC. Informed consent was obtained from all patients, and procedures involving human samples were approved by the Medical Ethical Committee of PKUCH. The PDX fragments were implanted subcutaneously into the right groin of a five‐week‐old male NOD/SCID mouse to amplify the tumor cells. The tumors were excised and subcutaneously inoculated into the bilateral groins of new NOD/SCID mice. When tumors reached 100 mm^3^, 25 mg kg^−1^ SPIO@1D5‐ICG was injected intravenously to test targeting effectiveness. To confirm the therapeutic effect of MHT, 25 mg kg^−1^ SPIO@1D5‐ICG or PBS were injected at the right and left groin, respectively, through four injections every three days. The mice were randomly divided into control and MHT groups for further studies.

### MPI Imaging

MPI was performed using the Momentum MPI scanner (Magnetic Insight, Inc., Alameda, CA, USA). For in vitro MPI, 1.0 to 2.0 mg mL^−1^ nanoparticles were placed in Eppendorf tubes for scanning (parameters: Z field of view (FOV), 4 cm; mode, default; and time estimate, 2 min). For in vivo imaging, all mice were anesthetized with pentobarbitone (50 mg kg^−1^, intraperitoneally) and underwent 2D MPI 0, 6, 12, 24, 48, and 72 h after injection (parameters: Z FOV, 12 cm; mode, default; time estimate, 2.8 min). All mice were analyzed by calculating the normalized MPI signals (NMS) as follows: NMS (%) = (TMS_x_/BMS_x_)/(TMS_0_/BMS_0_) × 100%, where TMS_x_ was the average MPI signal within the ROI of the tumor at each time after injection; and BMS_x_ was the background MPI signal, which was represented by the ROI of the muscle area at different times; BMS_0_ and TMS_0_ were the average MPI signals within the ROI of the background and tumor areas at the first time point, respectively. MPI data analysis was performed using the VivoQuant software.

### MHT In Vitro and In Vivo

The SPIO@1D5‐ICG magnetic hyperthermal effect was measured by placing the solution at the center of an induction coil to receive AMF (20A, 353KHZ, and 1.6KW) generated using an induction heating system (MSI Automation, USA). The magnetic heating properties and temperature changes were further characterized using an induction heating system fitted with a fiber thermocouple. The SAR of each sample was calculated from the temperature profiles using the following formula:^[^ ^56^ ^]^

(1) $$SAR=\frac{C_{H_{2}O}\times m_{H_{2}O}}{m_{Fe}}\;\times \frac{\Delta T}{\Delta t}$$

where C_H2O_ is the heat capacity of water (4.184 J/(g*K)), m_H2O_ is the mass of water in the sample, m_Fe_ is the mass of Fe in the sample, and $\frac{\Delta T}{\Delta t}$ is the slope of temperature increase during the first MHT time points. For the in vivo treatment with AMF, SPIO@1D5‐ICG‐subcutaneously injected xenografts were placed in the center of the induction coil to receive AMF (20A, 353 kHz, and 1.6KW) for 10 min. Intratumoural temperature was measured using an inserted fiber‐optic thermometer. Whole‐body infrared imaging of mice with exposed colonic tumors was performed using an infrared thermal camera (Fluke Ti27, USA) during AMF treatment. For in vitro treatment with AMF, MFC^CLDN18.2^ cells were previously incubated with SPIO@1D5‐ICG (60 µg mL^−1^) in T12.5 flasks for 12 h. Then, the T12.5 flasks were placed in the center of the induction coil to receive the AMF (20A, 353KHZ, 1.6KW) for 10 min.

### Flow Cytometry

Mice bearing MFC^CLDN18.2^ tumors were euthanized on day 21 post‐procedure, and ≈100 mg of the tissue was dissociated into a single cell suspension using 1 mg mL^−1^ solution of collagenase/dispase (MilliporeSigma) for 40 min at 37 °C. The cells were then filtered through a 70 µm strainer, and viability was assessed using a ViCell cell counter (BeckmanCoulter). Single‐cell suspensions were generated for each sample to ensure no bias in tissue selection. After Fc blocking, single‐cell suspensions containing 3 × 10^6^ cells/sample were stained using the appropriate fluorescent antibody cocktails (listed in Methods) and analyzed using flow cytometry (BD FACS Celesta/Novocyte Advanteon). Data analysis was performed using the FlowJo10.8.1 software.

### Characterization of ICD

MFC^CLDN18.2^ cells were incubated with 60 µg mL^−1^ SPIO@1D5‐ICG for 12 h and subsequently placed in the center of the induction coil to receive AMF (20A, 353KHZ, and 1.6KW) for 10 min. After 12 h of MHT, the cells were fixed with 4% paraformaldehyde on ice for 20 min and incubated with anti‐CRT antibody for 2 h at room temperature. The cells were further incubated with goat anti‐mouse IgG (Alexa Fluor 488) and imaged under CLSM to detect ecto‐CRT exposure. After the different treatments, MFC^CLDN18.2^ cells were collected and fixed with BD Cytofix/Cytoperm. Afterward, the cells were stained with an anti‐CRT‐specific antibody. Then, cells were washed with FACS buffer and incubated with a secondary antibody at 4 °C for 30 min. Finally, flow cytometry was used to detect CRT expression. To detect the extracellular HMGB1 and ATP, the culture supernatants were centrifuged at 13500 g at 4 °C for 10 min. HMGB1 release in the supernatant was measured using HMGB1 ELISA, and the levels of secreted and cytoplasmic ATP were quantified using an ATP bioluminescent assay kit according to the manufacturer's instructions.

### In Vitro BMDCs Maturation Study

BMDCs were isolated from the tibia and femur of 615 male mice (eight weeks old) according to established methods.^[^ ^48^ ^]^ RMPI 1640 culture medium supplemented with 10% FBS, 20 ng mL^−1^ GM‐CSF, and 10 ng mL^−1^ IL‐4 was used to induce BMDCs differentiation. For the in vitro BMDC maturation study, MFC^CLDN18.2^ cells were cultured overnight and treated with SPIO@1D5‐ICG and AMF, as described above. Next, BMDCs (5 × 10^5^ cells/well) were incubated with MHT‐treated MFC^CLDN18.2^ cells for 24 h. Finally, BMDCs were collected and stained with anti‐CD11c, anti‐CD80, and anti‐CD86 antibodies and detected using flow cytometry. Interleukin 12 (IL12p70), IL‐6, and TNF‐α secreted in the supernatant were measured using ELISA according to the manufacturer's instructions.

### Statistical Analyses

The diversity between subgroups was assessed using the chi‐square test, Wilcoxon test, unpaired two‐tailed t‐test, and one‐way or two‐way repeated‐measures ANOVA. Survival proportions were estimated using Kaplan–Meier analysis paired with the log‐rank test or COX regression model. Analyses and graphing were performed using R 4.1.1, SPSS 25.0, GraphPad Prism 9.5, Bio‐Render, Origin 2023, ImageJ 4.0 and FlowJo 10.8.1. Results were presented as the mean ± standard error of the mean.

## Conflict of Interest

The authors declare no conflict of interest.

## Supporting information



Supporting Information

## Data Availability

The data that support the findings of this study are available in the supplementary material of this article.
