# Supplementary material for: A CLDN18.2‐Targeted Nanoplatform Manipulates Magnetic Hyperthermia Spatiotemporally for Synergistic Immunotherapy in Gastric Cancer
Source: Adv Sci (Weinh). 2025 Feb 28;12(16):2413913. doi: 10.1002/advs.202413913 (PMC12021038; doi:10.1002/advs.202413913)
Supplement: Supplementary file 1 — Supporting Information [file ADVS-12-2413913-s001.docx]

Supporting Information

**A CLDN18.2-Targeted Nanoplatform Manipulates Magnetic Hyperthermia Spatiotemporally for Synergistic Immunotherapy in Gastric Cancer**

*Xueying Wang, Hui Hui, Jing Han, Ting Guo, Yiding Wang, Lin Meng, Cong Chen, Jie He, Xiaoyong Guo, Fuyu Zhong, Hong Du, Jie Tian,* Xiaofang Xing,* Yang Du,* Jiafu Ji**

Experimental Section

**1. Materials**

CLDN18.2 antibody 1D5 (US 20200207857A1); Mouse IgG (Solarbio, #SP031); SPIO-DSPE-PEG2000 (NANOEAST, #Mag3200); ICG-NHS ester (Ruixibio, #R-ICG-001); EDC (Invitrogen, #25952-53-8); LPS (Chemegen, #CX40002); IL-4 (Cell Signaling Technology, #3836); GM-CSF (Relia Tech, #101-M760); TNF-α ELISA kit (Invitrogen, #88-7324); IFN-γ ELISA kit (Servicebio; #GEM0006); IL-6 ELISA kit (Thermo Scientific, #BMS603HS); IL-12p70 ELISA kit (Servicebio; #GEM0009); HMGB1 ELISA kit (FineTest, #EM0382). Enhanced ATP Assay Kit (Beyotime; #S0027). Annexin V, FITC Apoptosis Detection Kit (DOJINDO, #AD10). Immunohistochemistry (IHC): ABCC2 (Proteintech, #24893-1-AP); ERCC1 (DAKO, #IR091); LYZ (Proteintech, #15013-1-AP); CD274 (Roche, #SP142); CD3 (Novo, NCL-CD3-565); ERBB2 (VENTANA, #4B5); PCNA (Millipore, #CBL407); CTNNB1 (DAKO, #IR702); Brd4 (Abcam, #Ab128874); CDH1 (DAKO, #IR059); PDCD1 (Cell marque, #315M-96); CD8 (Biocare, #CRM311C). Immunofluorescence analysis: HMGB1 (Abcam, #ab79823); Calreticulin (Abcam, # ab92516); CD8 (Cell Signaling Technology, #98941); CD11c (Cell Signaling Technology, #97585); PD1 (Cell Signaling Technology, #84651); PANCK (Absin, #123684); Hoechst 33342 (Invitrogen; #H1399); Dil (Beyotime, #C1036). Flow cytometry: CD3-PerCP-Cy5.5 (#560835); CD25-BV786 (#563701); CD8-BV605 (#564116); CD4-BV650 (#563875); CD45-BV510 (#563204); CD11c-APC-Cy7 (#561241); CD11b-BB515 (#564517); F4/80-PE (#565410); Ly6C-PE-Cy7 (#560593); PD1-PE-CF594 (#565024); PD-L1-BV421 (#568923); CD86-BV711 (#563158); CD206-APC (#141708); CD80-PE (#104707); CD86-PE-Cy7 (#105013); and Alexa Fluor® 700 (#557943) were obtained from BD Biosciences and BioLegend.

**2. Cell lines and cell culture**

Human GC cell lines (AGS, BGC823, HGC27, MKN45, MKN28, MGC803, SGC7901, and N87), mouse GC cell line MFC, and normal gastric epithelial cell line GES-1 were obtained from Cell Bank of Chinese Academy of Sciences, grown in high-glucose Dulbecco’s Modified Eagle’s Medium (DMEM; GIBCO, Carlsbad, USA) supplemented with 10% fetal bovine serum (FBS; GIBCO, Carlsbad, USA) and 1% penicillin/streptomycin. All the cell lines were maintained at 37 °C in a humidified 5% CO_2_ incubator.

**3. Immunofluorescence and subcellular colocalization**

Cells were seeded in confocal dishes and grown to 90% confluence. After washing twice with PBS, cells were incubated with 2 μg/mL CLDN18.2 antibody (1D5) and Hoechst 33342 for 45 min at 37 °C followed by probing with an Alexa Fluor 488-conjugated anti-mouse antibody (CST, 4408s) for 45 min at room temperature in the dark. A confocal laser scanning microscopy (CLSM, Dragonfly 200, Andor, Shenzhen, China) was used to acquire images of fixed settings (40× lens) and exposure time at room temperature. The Pearson correlation coefficient (PCC) value was calculated using the analyze-colocalization-color 2 plugin in Image J software.

**4. *Ex vivo* histology, Prussian blue staining, and liver/kidney function evaluation.**

After *ex vivo* FMI and MPI, the major organs and tumors were immersed in 10% formalin and embedded in paraffin. Haematoxylin and eosin (H&E) and Prussian blue staining were conducted to analyze the biotoxicity and distributions of nanoparticles. CLDN18.2 was stained with a 1D5 antibody. The blood samples were collected and stored for 1 h at room temperature and centrifuged at 3,500 rpm for approximately 15 min to separate serum samples for biochemical examination and ELISA analysis.

**5. FMI imaging**

For *in vitro* FMI, nanoparticles at different concentrations from 0.3125 to 25.00 mg/mL were placed into 96-well plates, respectively, for capturing by IVIS. Isoflurane-anesthetized mice underwent FMI after intravenous injection of nanoparticles (IVIS spectrum). Images were captured before injection and after 1 h, 2 h, 4 h, 8 h, 12 h, 24 h, and 48 h at excitation and emission wavelengths of 780 and 831 nm. After the in vivo image acquisition, the mice of each group were euthanized to obtain the major organs (heart, liver, spleen, lung, kidney, and tumor) for *ex vivo* imaging. The Living Image 4.4 Software (PerkinElmer, Waltham, MA, USA) was used to analyze the results quantitatively. All mice were analyzed by calculating the normalized fluorescence intensity (NFI) as follows: NFI (%) = (TFI_x_ / BFI_x_)/ (TFI_0_ / BFI_0_) ×100%, where TFI_x_ is the average fluorescence intensity of the ROI of the tumor at each time point after injection, and BFI_x_ is the background fluorescence intensity, which is represented by the ROI of the muscle area at different time points; BFI_0_ and TFI_0_ represent the average fluorescence intensity of the background and tumor areas of the ROI at the first time point after nanoparticles injection, respectively.

**6. MRI imaging**

All mice were scanned using a 1.0 T M3TM magnetic resonance imaging (MRI) imager (Aspect Imaging, Shoham, Israel) with a 38 mm body coil. The mice were anesthetized using pentobarbitone (50 mg/kg, IP). MRI images were captured using a T2-Turbo RARE sequence (parameters: TR, 5557 ms; TE, 66.61 ms; FOV, 30 × 60 mm; slice orientation, coronal and transverse; and thickness, 0.8 mm).

**7. Multiplex Immunohistochemistry**

Immunofluorescence staining was performed on Intellipath Automated IHC Stainer (Biocare). Peroxidase block with Bloxall (Vector, SP-6000), followed by two washes in TBST and blocking with 3% Donkey Serum. Primary antibody incubations were carried out sequentially for 1 h followed by two washes in TBST and incubation with corresponding secondary antibody, anti-Rat HRP Polymer (Cell IDX, 2AH-100). The tissues were washed twice in TBST, developed using Tyramide Reagent, and washed ×2 in deionized H2O. Tissues were counterstained with DAPI (1 μg/mL). The slides were imaged using a Zeiss Axio Scan Z1 slide scanner with a 20×0.8 NA objective, Colibri7 light source, and high-efficiency filter sets. All slide images were analyzed using QuPath 3.0 software with individual fluorescence channels (DAPI, 570, 620, 520, and 700) set at constant thresholds across pictures and groups.

**Supplementary Figures**

**
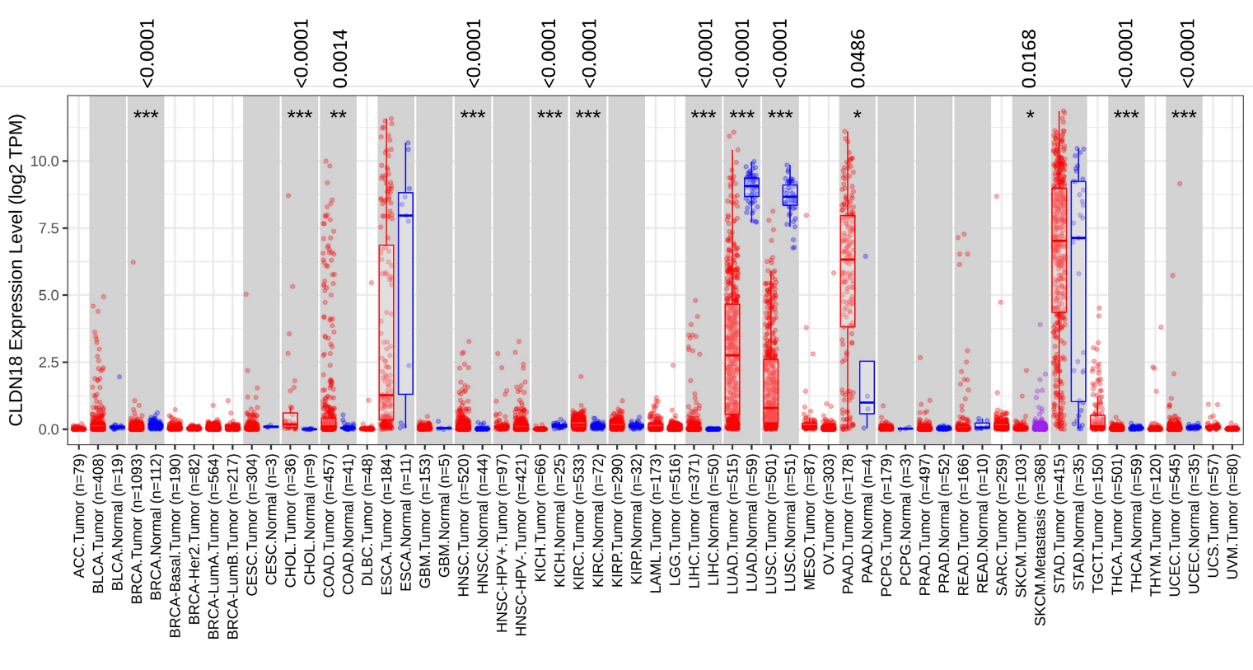
**

**Figure S1.** CLDN18 expression in pan-cancer and normal tissues in the TIMER 2.0 database. The differential mRNA expression level of CLDN18 between tumor and adjacent normal tissues across all TCGA tumors. Statistical significances were computed by a Wilcoxon test.

**
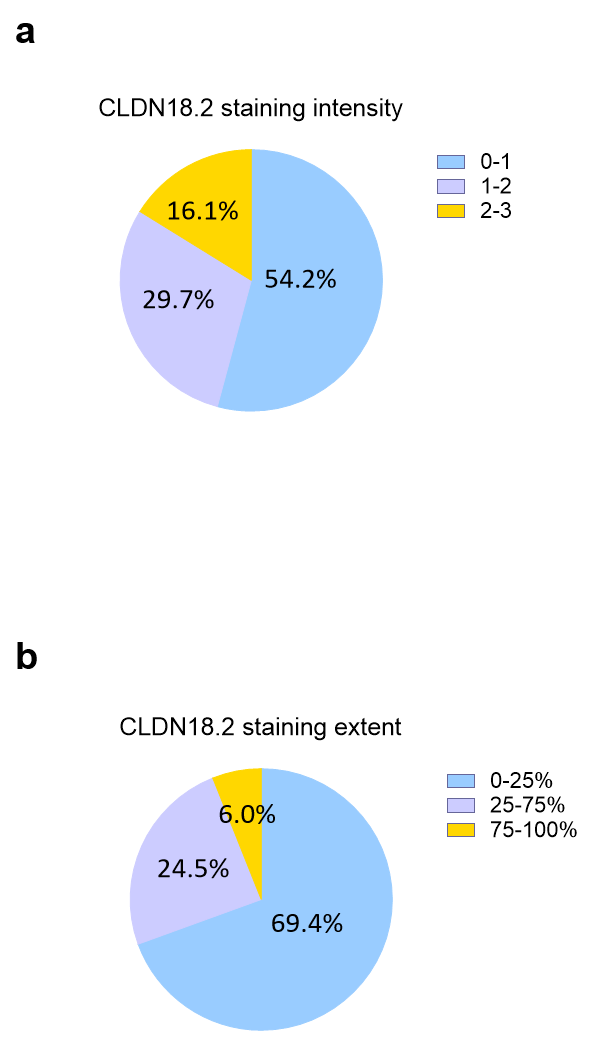
**

**Figure S2.** Quantification of CLDN18.2 underlying immunohistochemistry (IHC) analysis. a) Percentage of CLDN18.2 staining intensity analyzed in PKUCH TMA samples. b) Percentage of CLDN18.2 staining extent analyzed in PKUCH TMA samples.


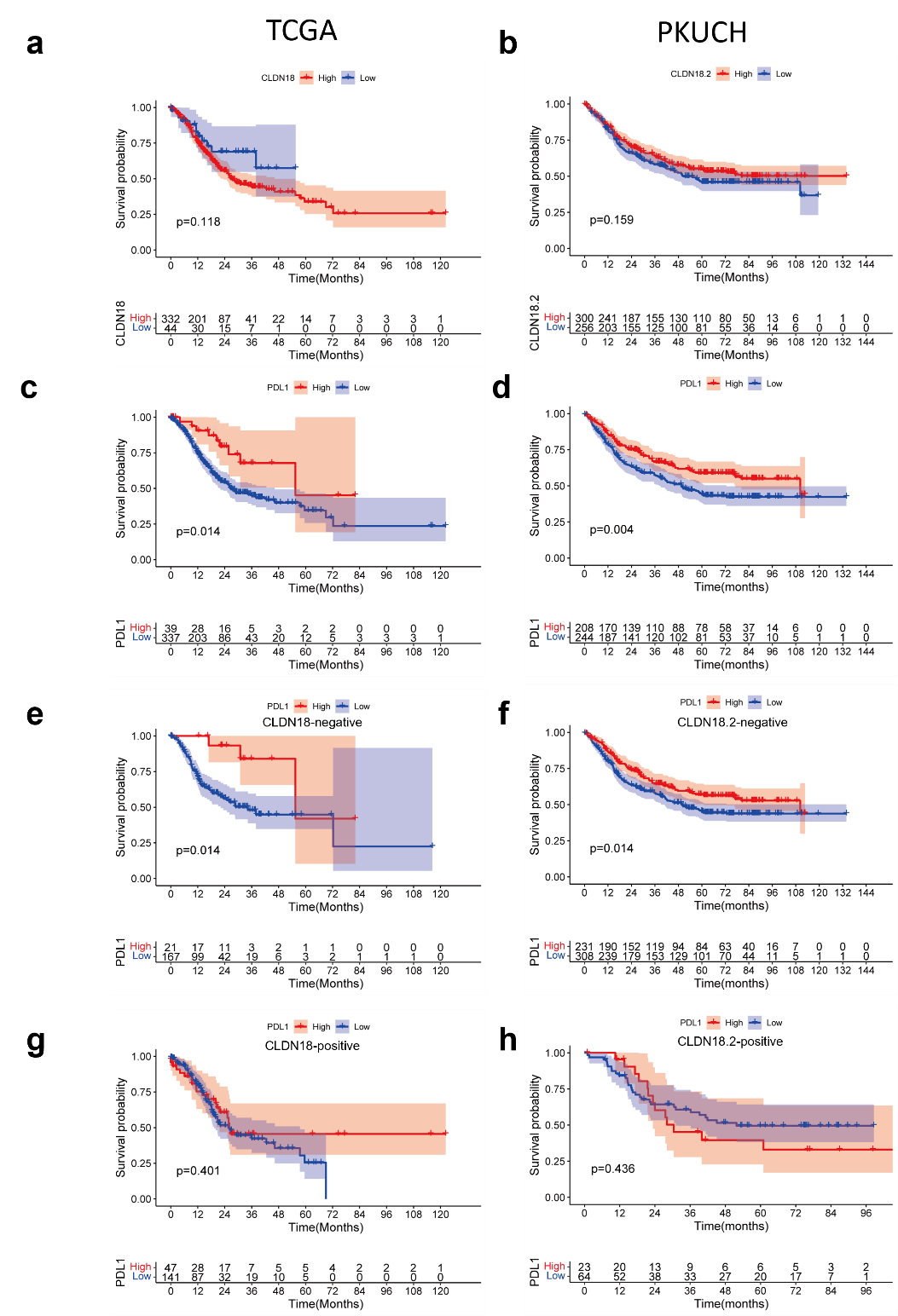


**Figure S3.** The optimal cut-off value defines Kaplan-Meier curves of overall survival in PKUCH or TCGA databases. a, b) Kaplan-Meier curves of overall survival in PKUCH (a) or TCGA (b) databases with high or low CLDN18.2/CLDN18 expression. c, d) Kaplan-Meier overall survival curves in PKUCH (c) or TCGA (d) databases with high or low PD-L1/CD279 expression. e, f) In CLDN18.2 negative subgroups, Kaplan-Meier overall survival curves in PKUCH (e) or TCGA (f) databases with high or low PD-L1/CD279 expression. g, h) In CLDN18.2 positive subgroups, Kaplan-Meier overall survival curves in PKUCH (g) or TCGA (h) databases with high or low PD-L1/CD279 expression. Statistical significances were calculated via a log-rank test.


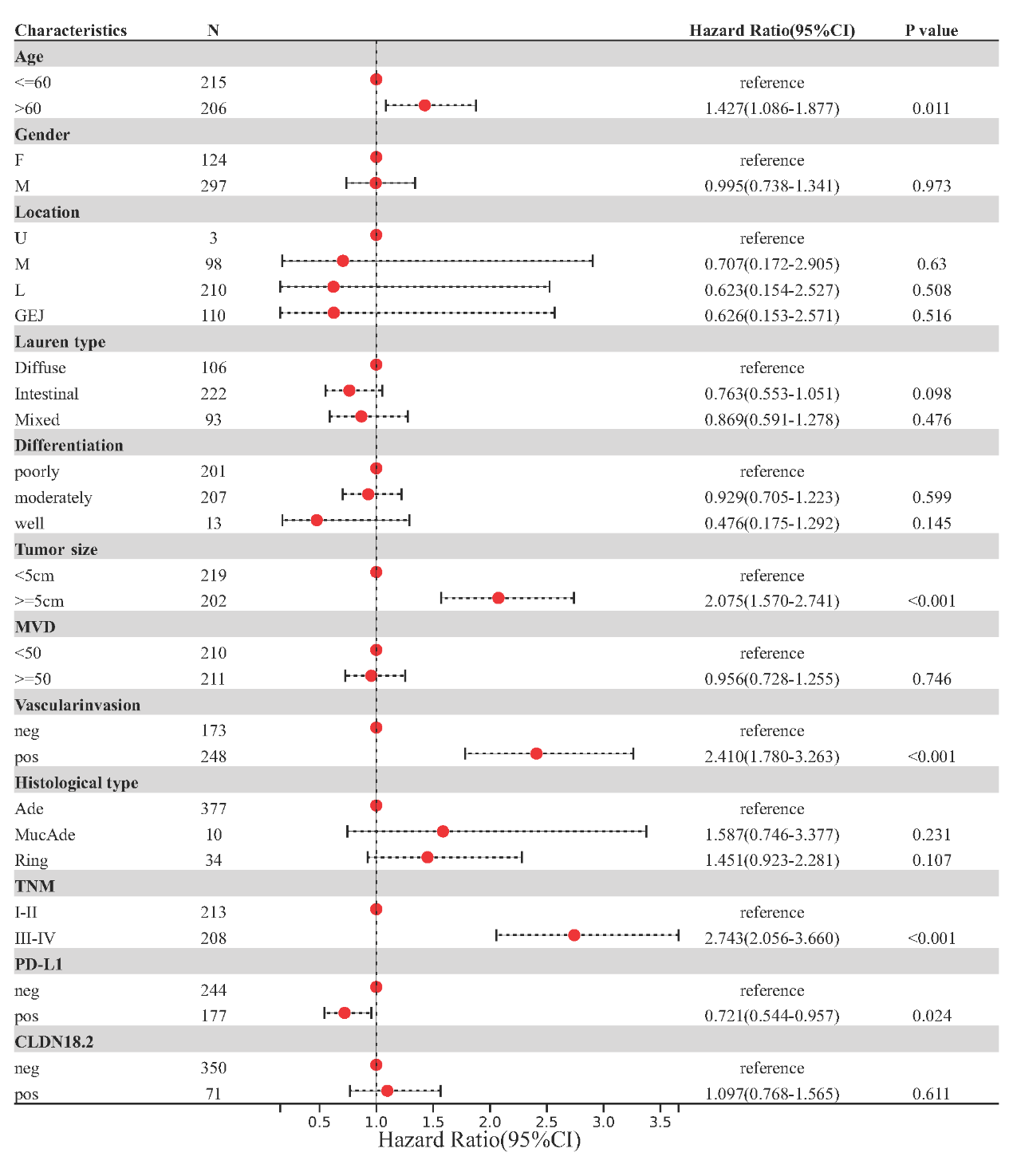


**Figure S4.** Forest plot of 12 prognostic-related clinicopathological features through univariate Cox analysis. CI: confidence interval.


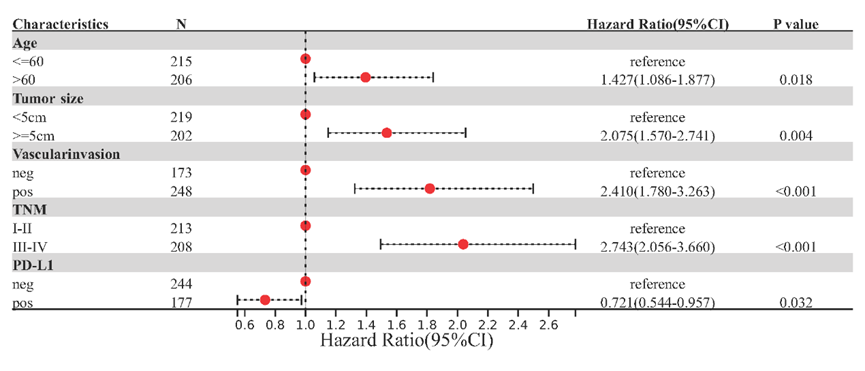


**Figure S5.** Forest plot of 5 prognostic-related clinicopathological features through multivariate Cox analysis. CI: confidence interval.


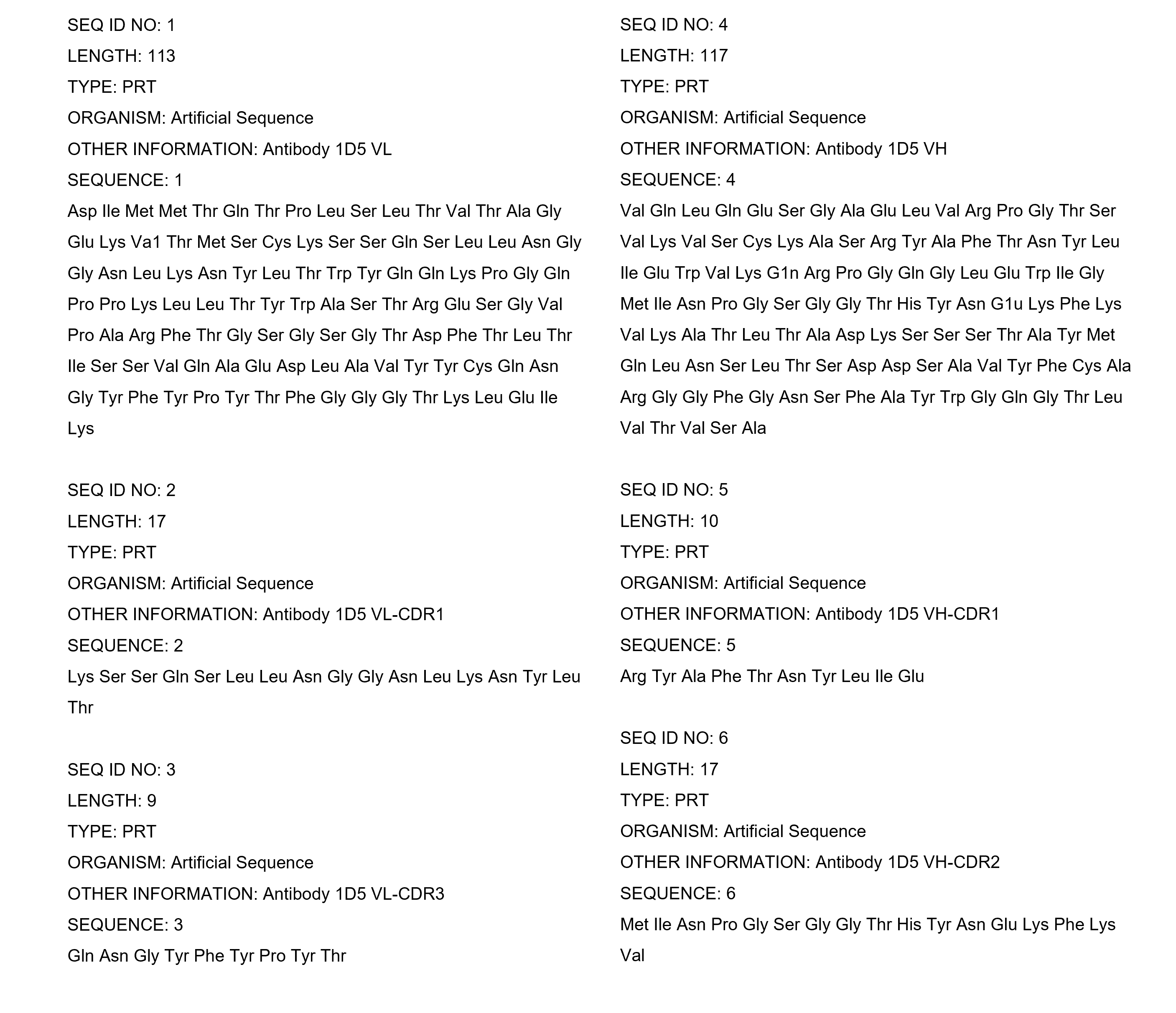


**Figure S6.** CLDN18.2 antibody 1D5 information. 1D5 comprises two light chain variable domains (VLs) comprising the amino acid sequence of SEQ ID NO: 1 and two heavy chain variable domains (VHs) comprising the amino acid sequence of SEQ ID NO: 4. Representative CDR sequences were shown at SEQ ID NO: 2-3 in VLs and SEQ ID NO: 5-6 in VHs.

**
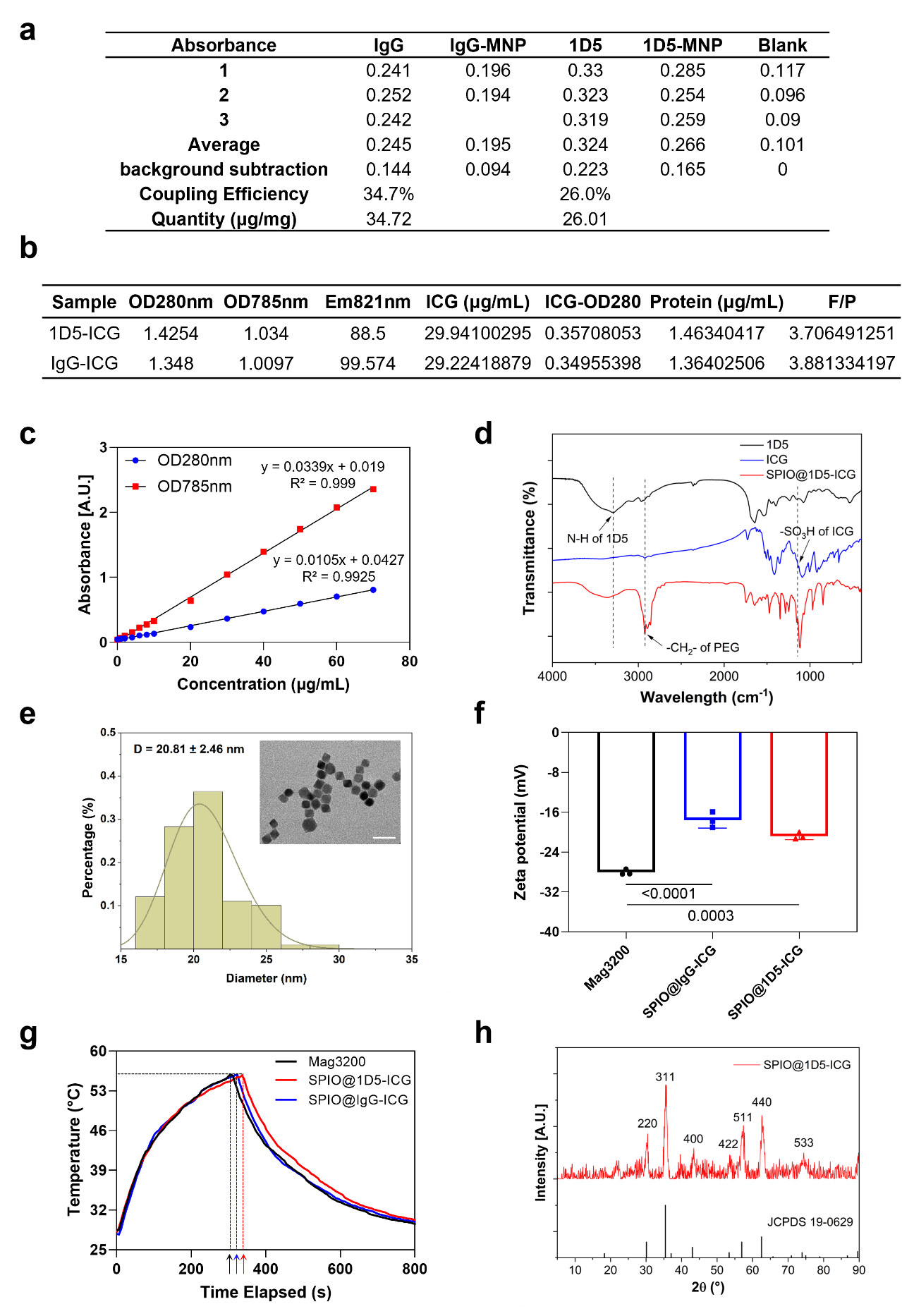
**

**Figure S7.** Supplemental characteristics of SPIO@1D5-ICG. a) bicinchoninic acid (BCA) analysis of synthesized nanoparticles. b) BCA analysis of 1D5-ICG. MNP: magnetic nanoparticles; OD: optical density; Em: Emission wavelength; F/P: Fluorophore-to-Protein ratio. c) Standard curve of UV absorption OD value of 1D5-ICG at 774 nm and 280 nm. d) Fourier transform infrared (FTIR) spectra of 1D5, ICG, and SPIO@1D5-ICG. e) Representative transmission electron microscopy (TEM) images of SPIO@1D5-ICG (inset: size distribution of SPIO analyses). Scale bars = 50 nm. f) Zeta potential of SPIO@1D5-ICG. Statistical significances were calculated using the one-way ANOVA and Dunnett multiple comparisons test. g) Magnetothermal heating/cooling curves of Mag3200, SPIO@1D5-ICG, and SPIO@IgG-ICG (1 mg/mL) under the AMF (20A, 353KHZ, 1.6KW). The arrows of different colors represent the time points of AMF turn-off in different groups. h) X-ray Diffraction of SPIO@1D5-ICG compared with standard Fe_3_O_4_ sample (JCPDS 19-0629).


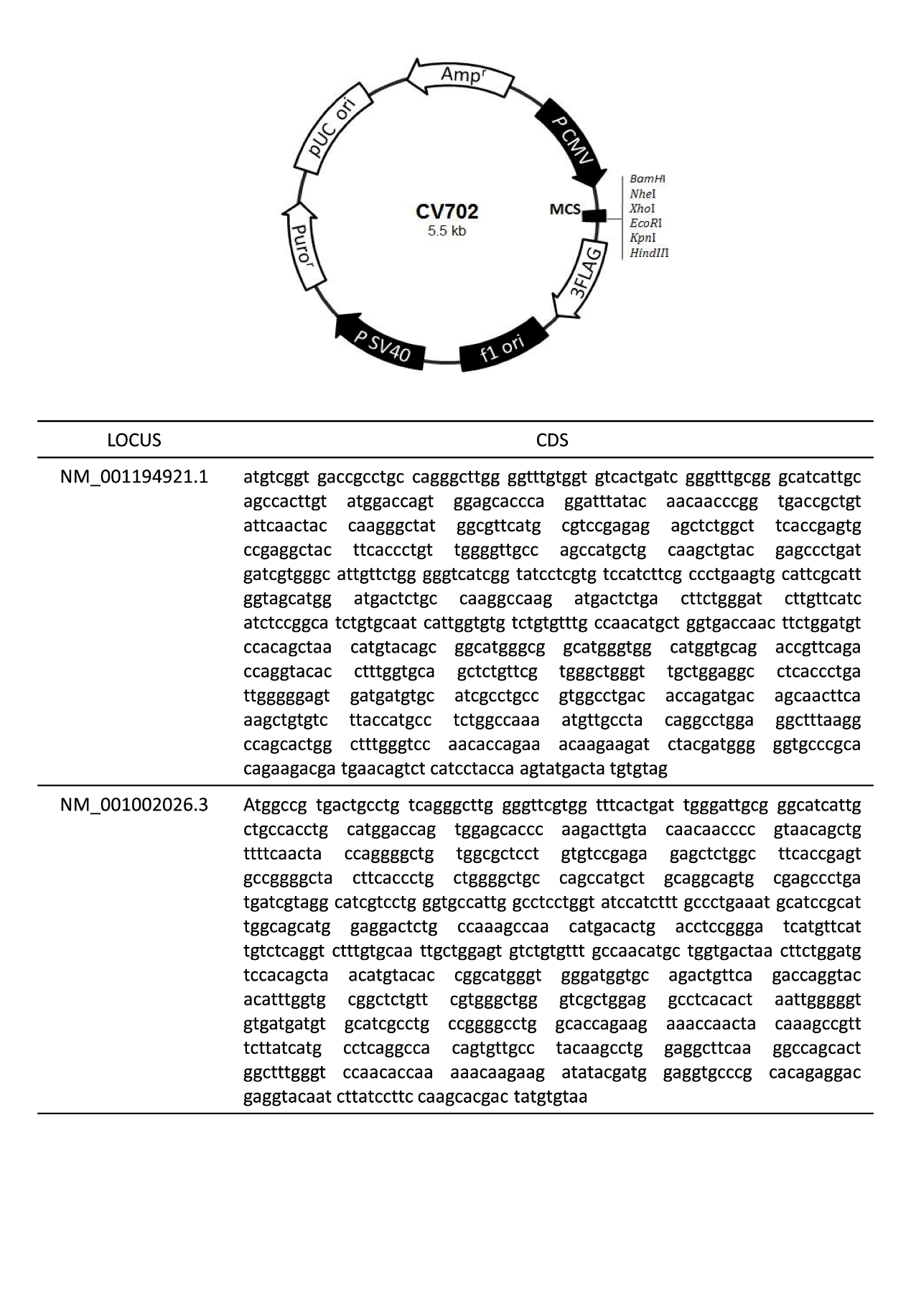


**Figure S8.** Plasmid information in this study.


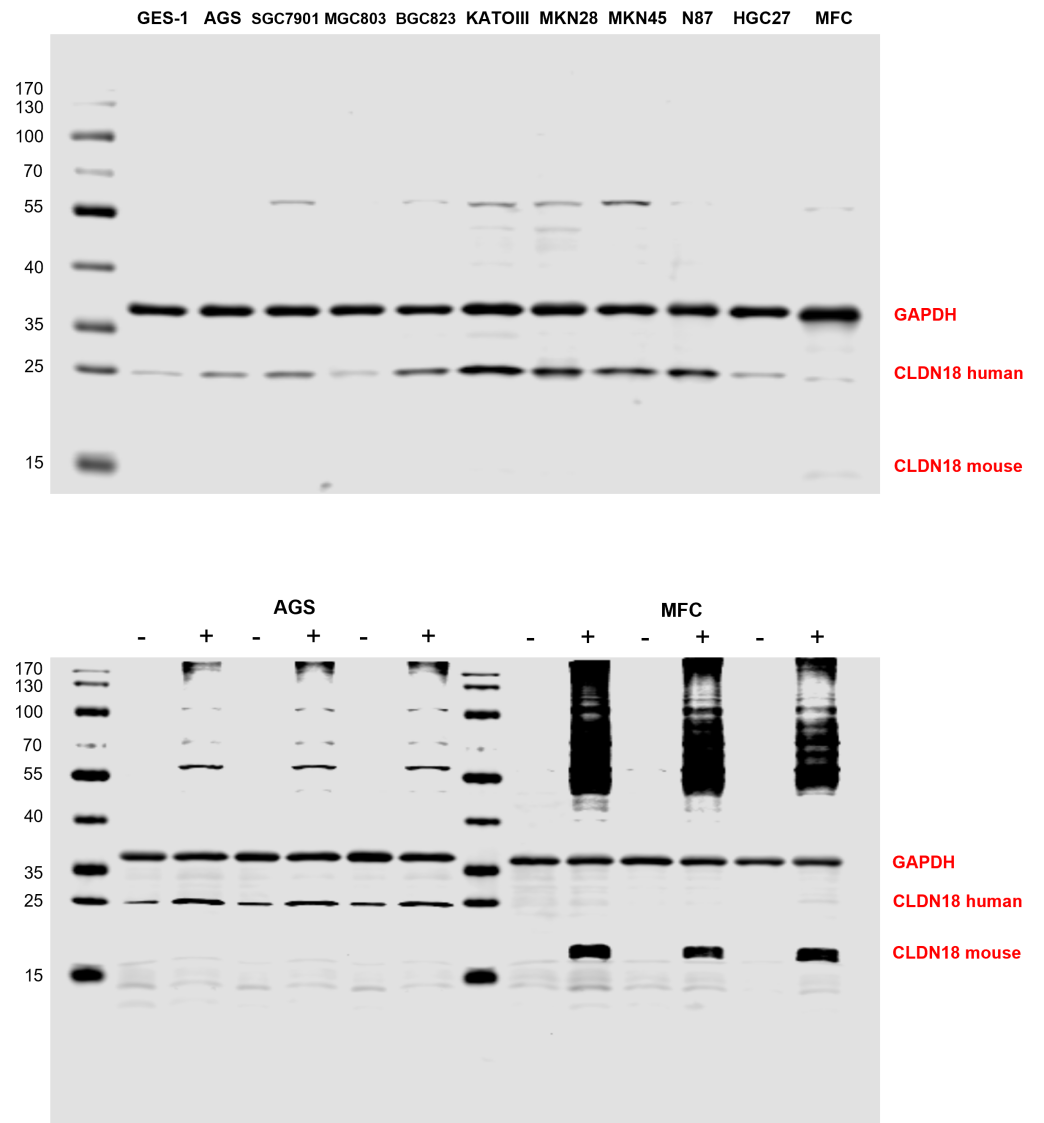


**Figure S9.** Full gel scan images. a) Uncropped blot of Figure 3f. b) Uncropped blot of Figure 3g.


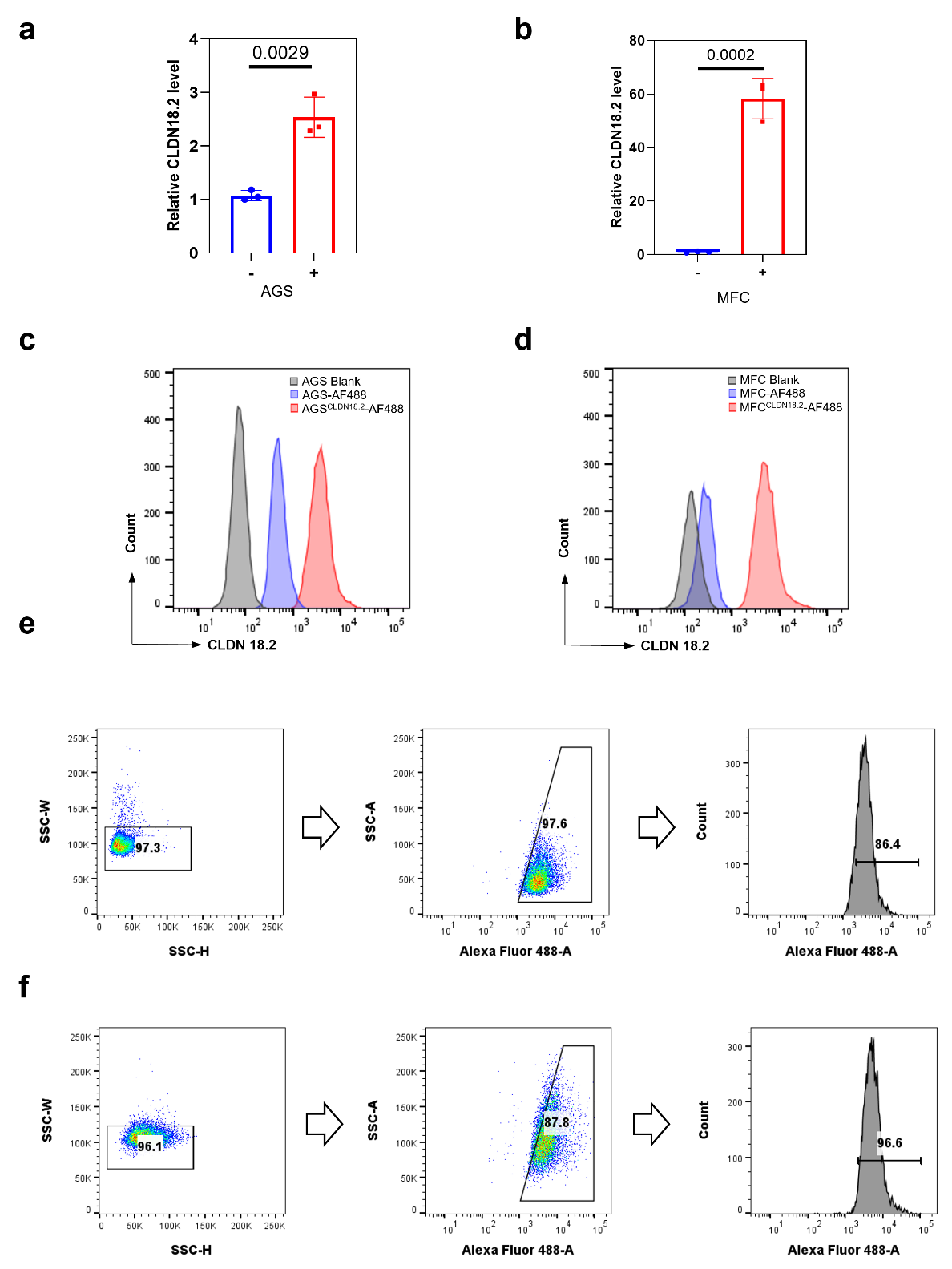


**Figure S10.** CLDN18.2 overexpression in AGS and MFC cells. a, b) Quantitative analysis of WB in AGS/AGS^CLDN18.2^ cells (a) and MFC/MFC^CLDN18.2^ cells (b). c, d) Flow cytometry histogram of AGS/AGS^CLDN18.2^ cells (c) and MFC/MFC^CLDN18.2^ cells (d) incubated with the anti-CLDN18.2 antibody 1D5 and secondary AF488 antibodies. e, f) Representative FACS gating strategy of flow cytometry for detecting the percentage of CLDN18.2 overexpressed AGS (e) and MFC (f) cells. Statistical significance was calculated by the unpaired two-tailed Student’s t-test (a, b).


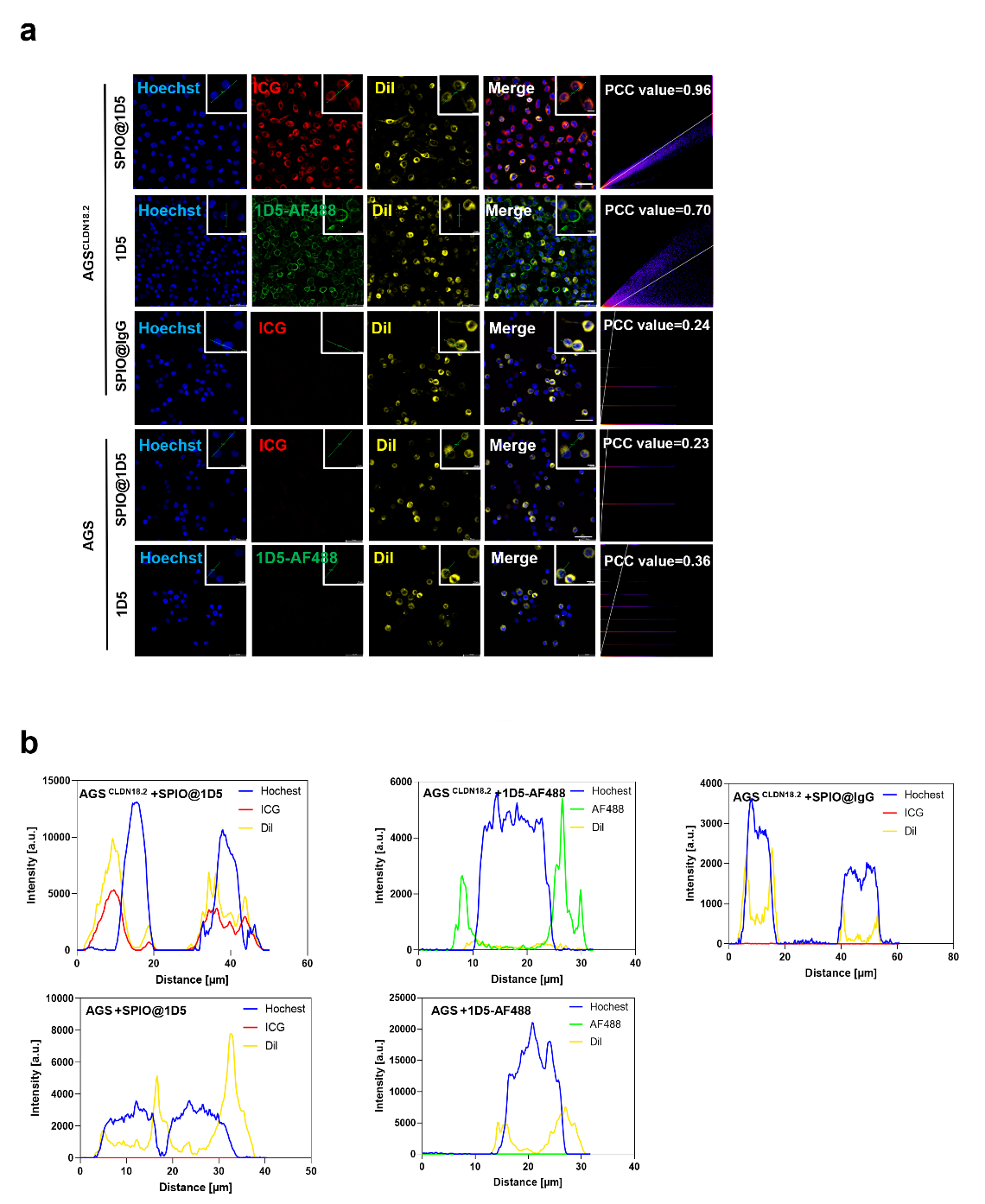


**Figure S11.** Immunofluorescence of 1D5-targeted nanoparticle in AGS^CLDN18.2^ and AGS cell lines. a) Confocal laser scanning microscopy images of AGS^CLDN18.2^ cells incubated with SPIO@1D5-ICG, 1D5-AF488 and SPIO@IgG-ICG, and AGS cells incubated with SPIO@1D5-ICG/1D5 for 2 h at a Fe concentration of 60 μg/mL (scale bar: 50 μm; scale bar in magnified pictures: 10 μm). Colocalization of internalized nanoparticles (red) or 1D5 (green) with Cell membrane staining (Dil, yellow) colocalization. Pearson’s correlation coefficient (PCC) value was calculated by the analyze-colocalization-color 2 plugin in the software Image J. b) The corresponding quantification results of fluorescent intensity of the line scanning profiles were provided for each group. Red lines represent ICG. Green lines represent AF488. Yellow lines represent Dil. Blue lines represent DAPI.


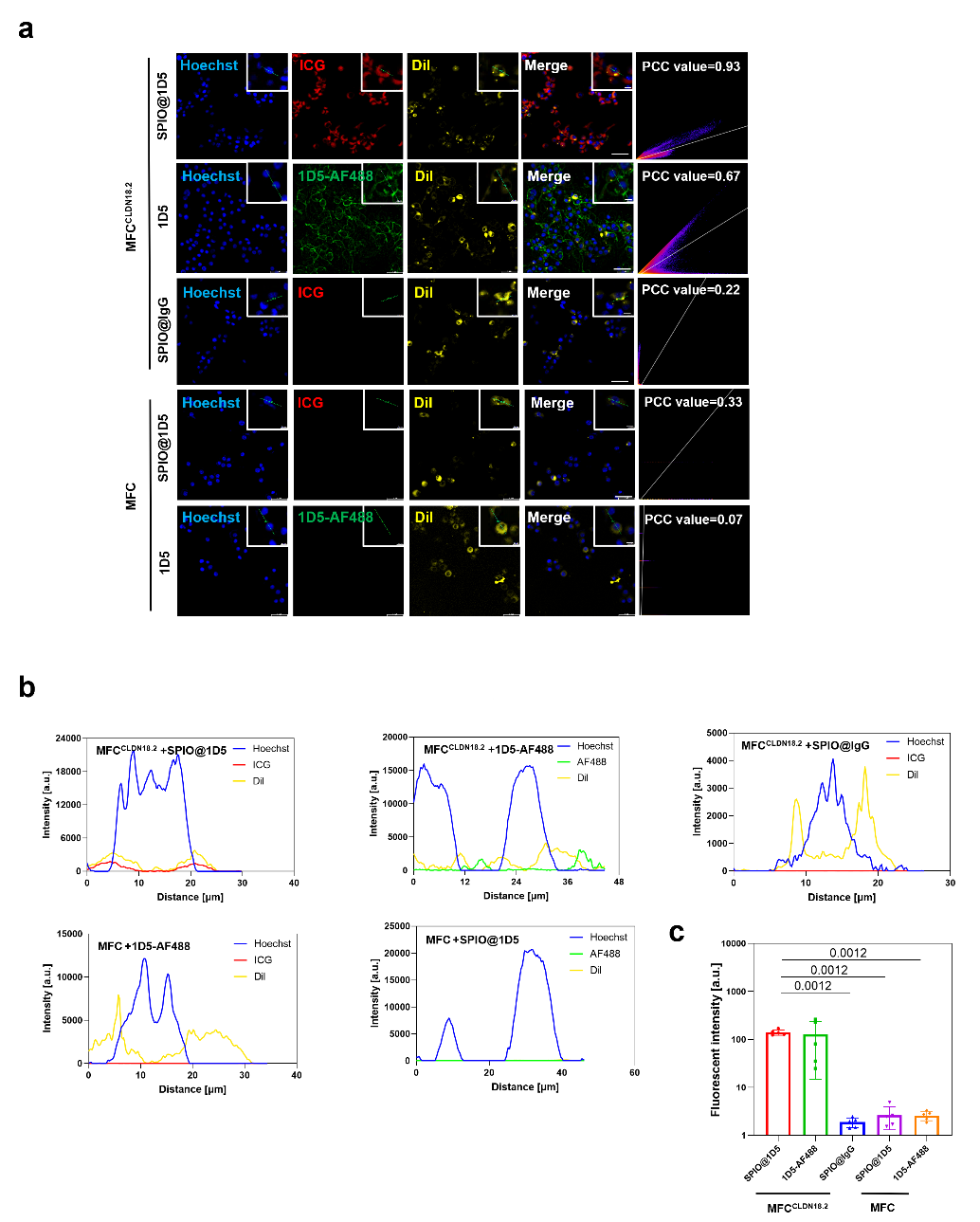


**Fig. S12.** Immunofluorescence of 1D5-targeted nanoparticle in MFC^CLDN18.2^ and MFC cell lines. a) Confocal laser scanning microscopy images of MFC ^CLDN18.2^ cells incubated with SPIO@1D5-ICG, 1D5-AF488 and SPIO@IgG-ICG, and MFC cells incubated with SPIO@1D5-ICG/1D5 for 2 h at a Fe concentration of 60 μg/mL (scale bar: 50 μm; scale bar in magnified pictures: 10 μm). Colocalization of internalized nanoparticles (red) or 1D5 (green) with Cell membrane staining (Dil, yellow) colocalization. Pearson’s correlation coefficient (PCC) value was calculated by the analyze-colocalization-color 2 plugin in the software Image J. b) The corresponding quantification results of fluorescent intensity of the line scanning profiles were provided for each group. Red lines represent ICG. Green lines represent AF488. Yellow lines represent Dil. Blue lines represent DAPI. c) Quantitative analysis of fluorescence intensity in SPIO@1D5-ICG, 1D5-AF488, SPIO@IgG-ICG incubated MFC^CLDN18.2^ cells, as well as in SPIO@1D5-ICG and 1D5-AF488 treated MFC cells.


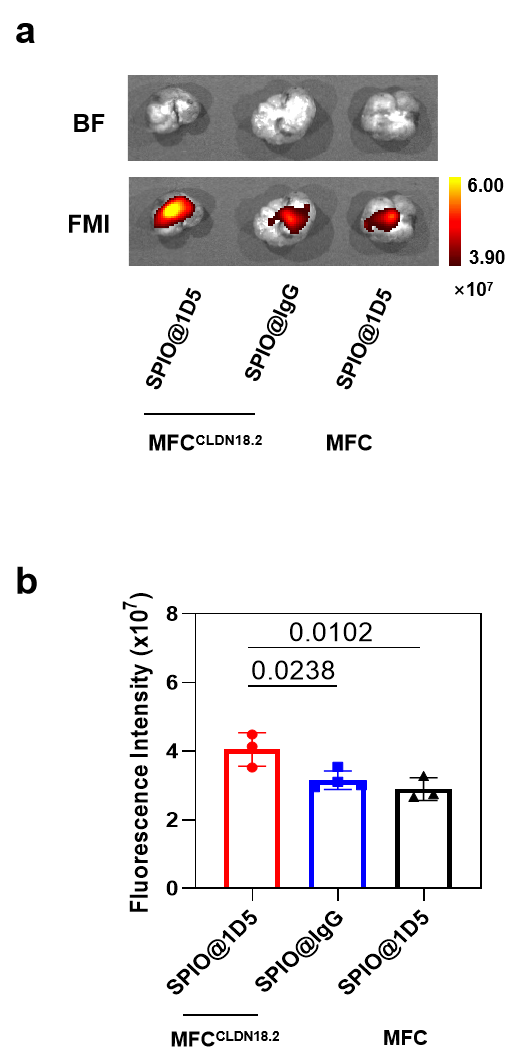


**Figure S13.** *Ex vivo* FMI distribution of 1D5@SPIO in tumors in MFC^CLDN18.2^ allografts. a) Fluorescence images of *ex vivo* MFC^CLDN18.2^ tumors. b) Quantitative comparison of fluorescence intensity in tumors. Data are expressed as means ± SD (n = 3). Statistical significances were calculated via a one-way ANOVA test.


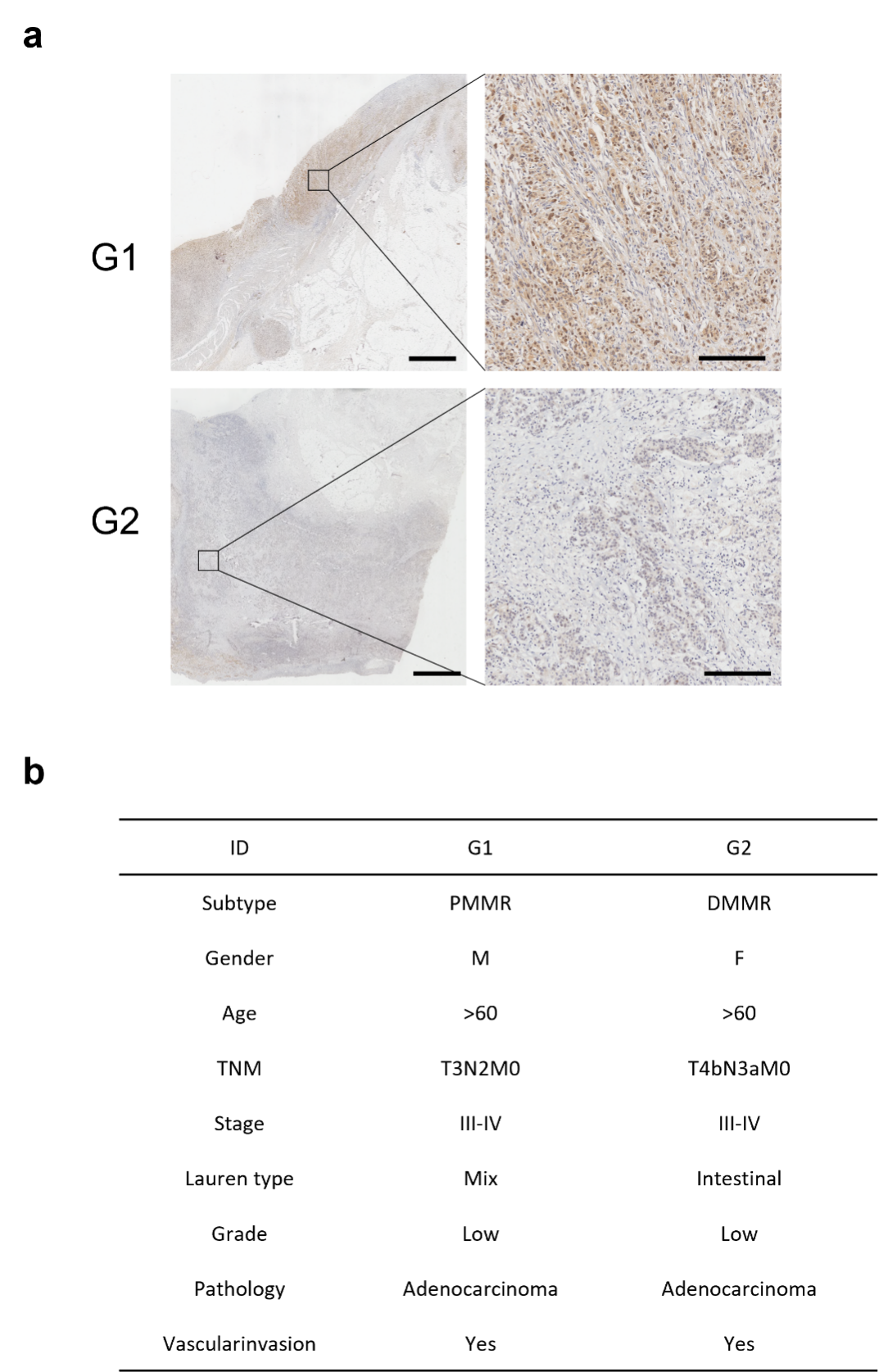


**Figure S14.** Tumour engraftment PDX models with different CLDN18.2 expressions. a) Immunohistochemistry of PDX models. Scale bar: 2 mm; scale bar in magnified pictures: 200 μm. b) Clinicopathological features of these samples.


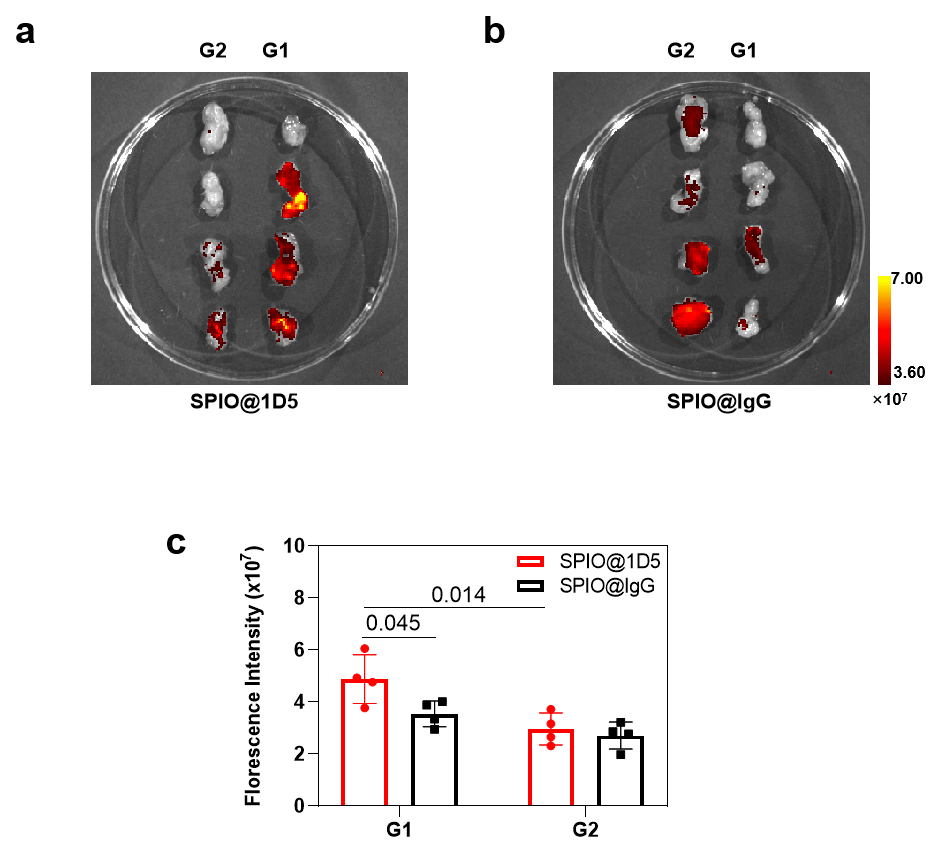


**Figure S15.** *Ex vivo* FMI of the distribution of SPIO@1D5 in tumors in PDX models. a, b) Fluorescence images of *ex vivo* PDX tumors after injection with SPIO@1D5 (a) or SPIO@IgG (b). c) Quantitative comparison of fluorescence intensity in G1 and G2 tumors. Statistical significances were calculated via the unpaired two-tailed Student’s t-test.


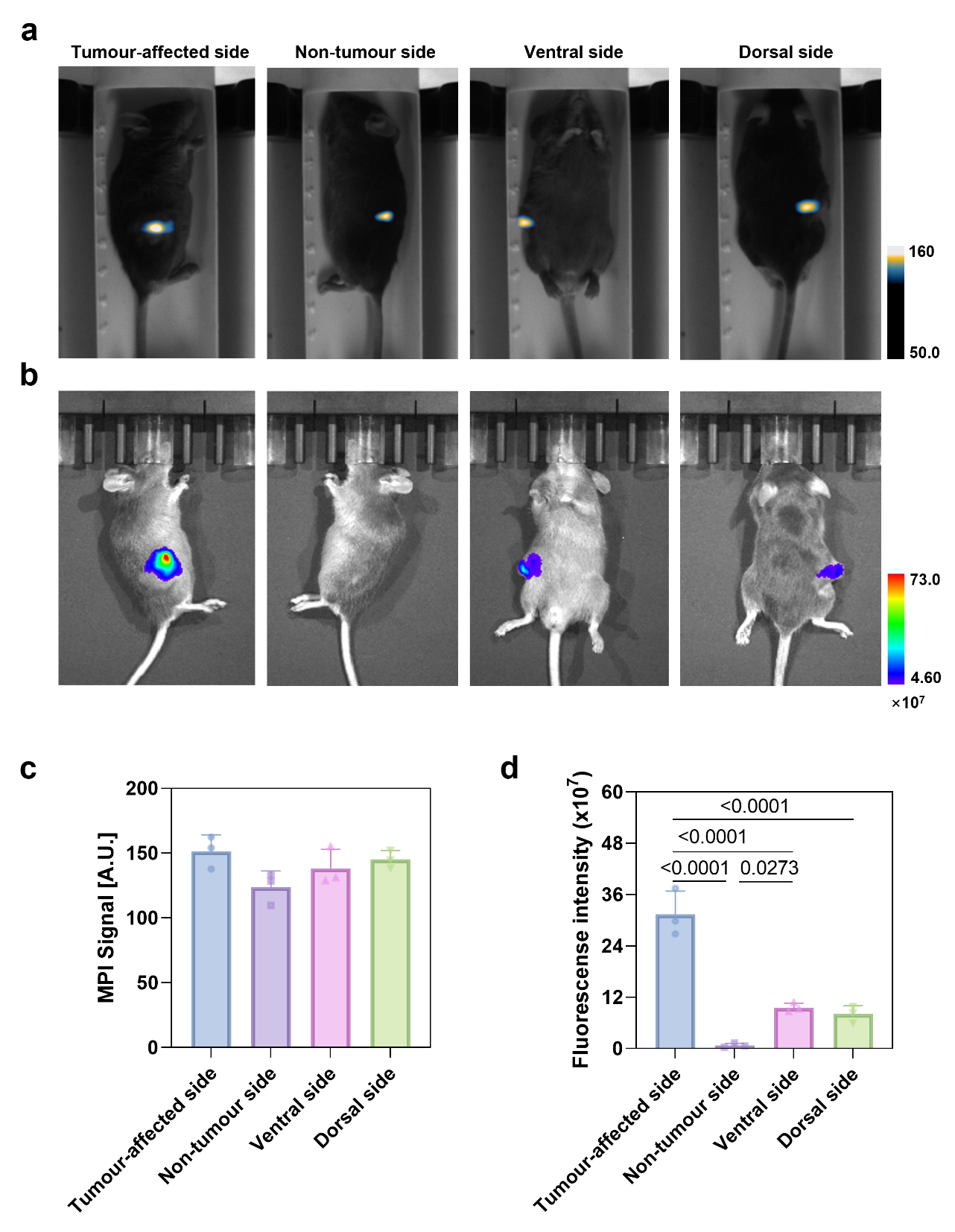


**Figure S16.** MPI and FMI signals of 615 mouse subcutaneous MFC^CLDN18.2^-bearing mice at different positions. a) MPI images of SPIO@1D5 intratumorally injected MFC^CLDN18.2^-bearing 615 mice. b) FMI images of SPIO@1D5 intratumorally injected MFC^CLDN18.2^-bearing 615 mice. c, d) Quantitative analysis of MPI (c) and FMI (d) signals at different positions. Data are expressed as means ± SD (n = 3). Statistical significances were calculated via one-way ANOVA and Tukey multiple comparisons test.


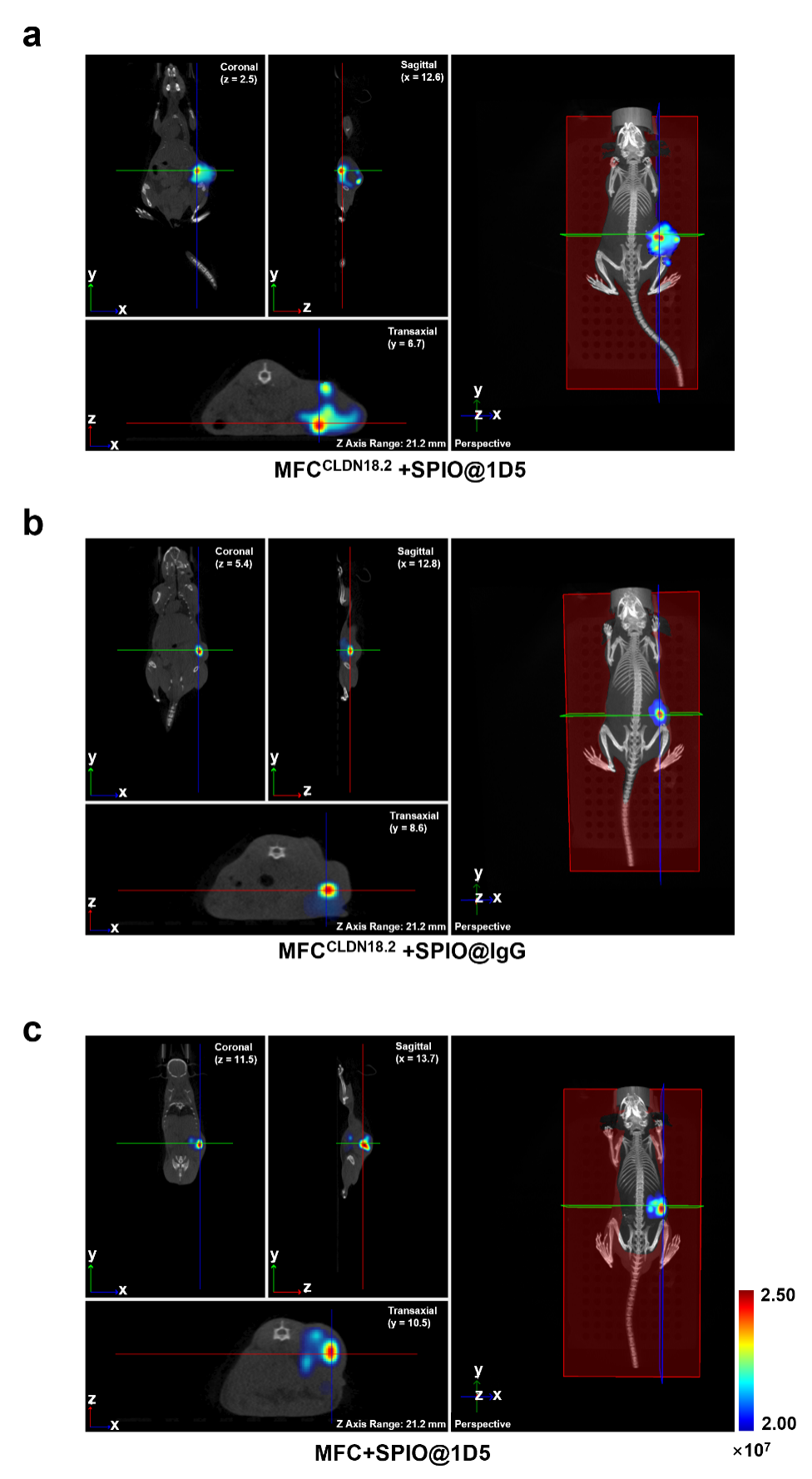


**Figure S17.** Representative FMI-CT 3D reconstruction of MFC/MFC^CLDN18.2^-bearing 615 mice after intratumoral injection with SPIO@1D5 / SPIO@IgG for 12 h. Left panel clockwise: coronal, sagittal, and trans-axial views.


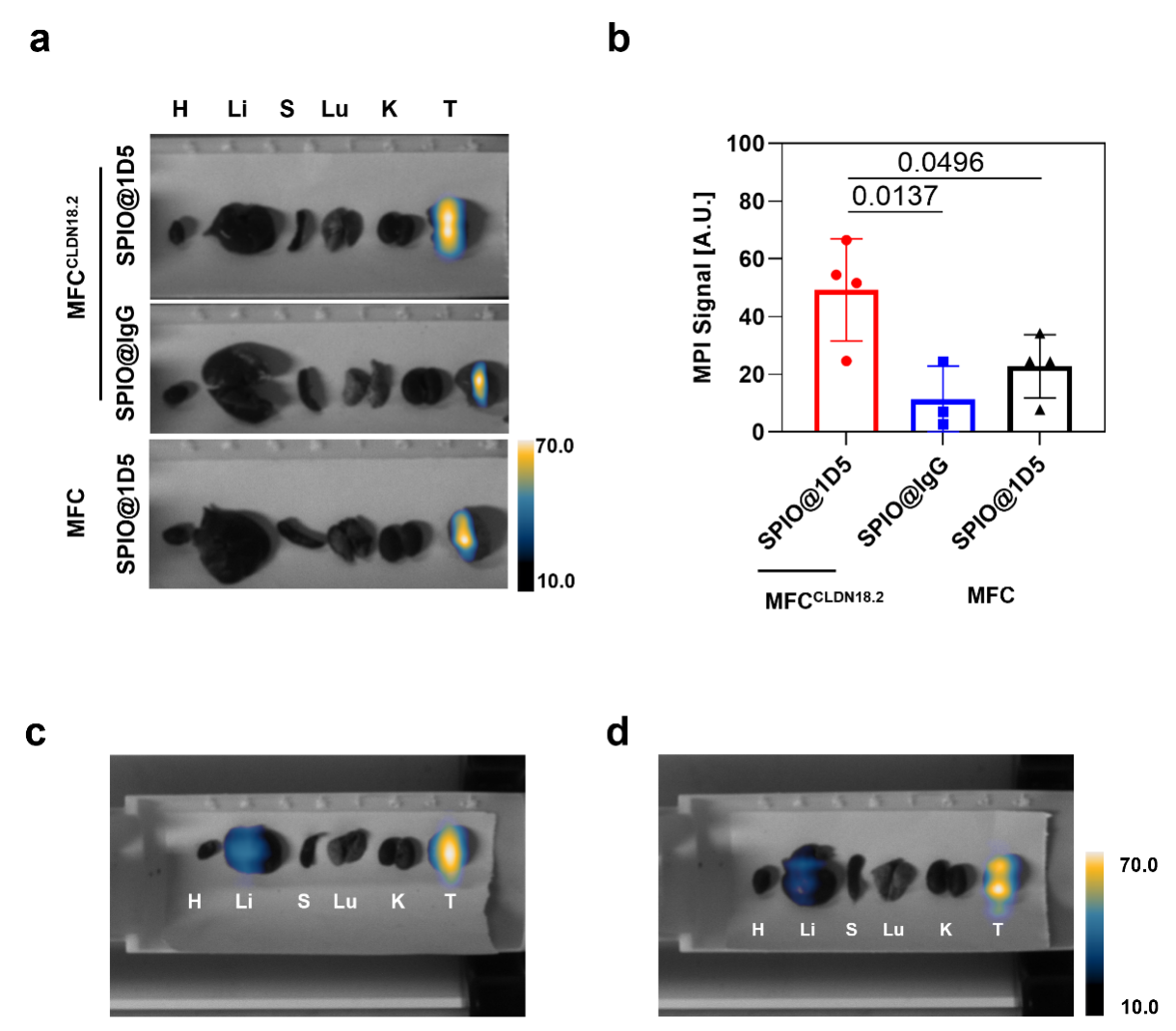


**Figure S18.** *Ex vivo* MPI of the distribution of SPIO@1D5 in tumors and major organs in MFC^CLDN18.2^ allografts. a) *Ex vivo* MPI images of resected tumors and major organs from the subcutaneous model at 72 h. H: heart; Li: liver; S: spleen; Lu: lung; K: kidney; T: tumor. b) Quantitative comparison of MPI signal in tumors. Data were expressed as means ± SD (n = 4). c, d) SPIO@1D5 treated MFC group could detect MPI signal in the liver.


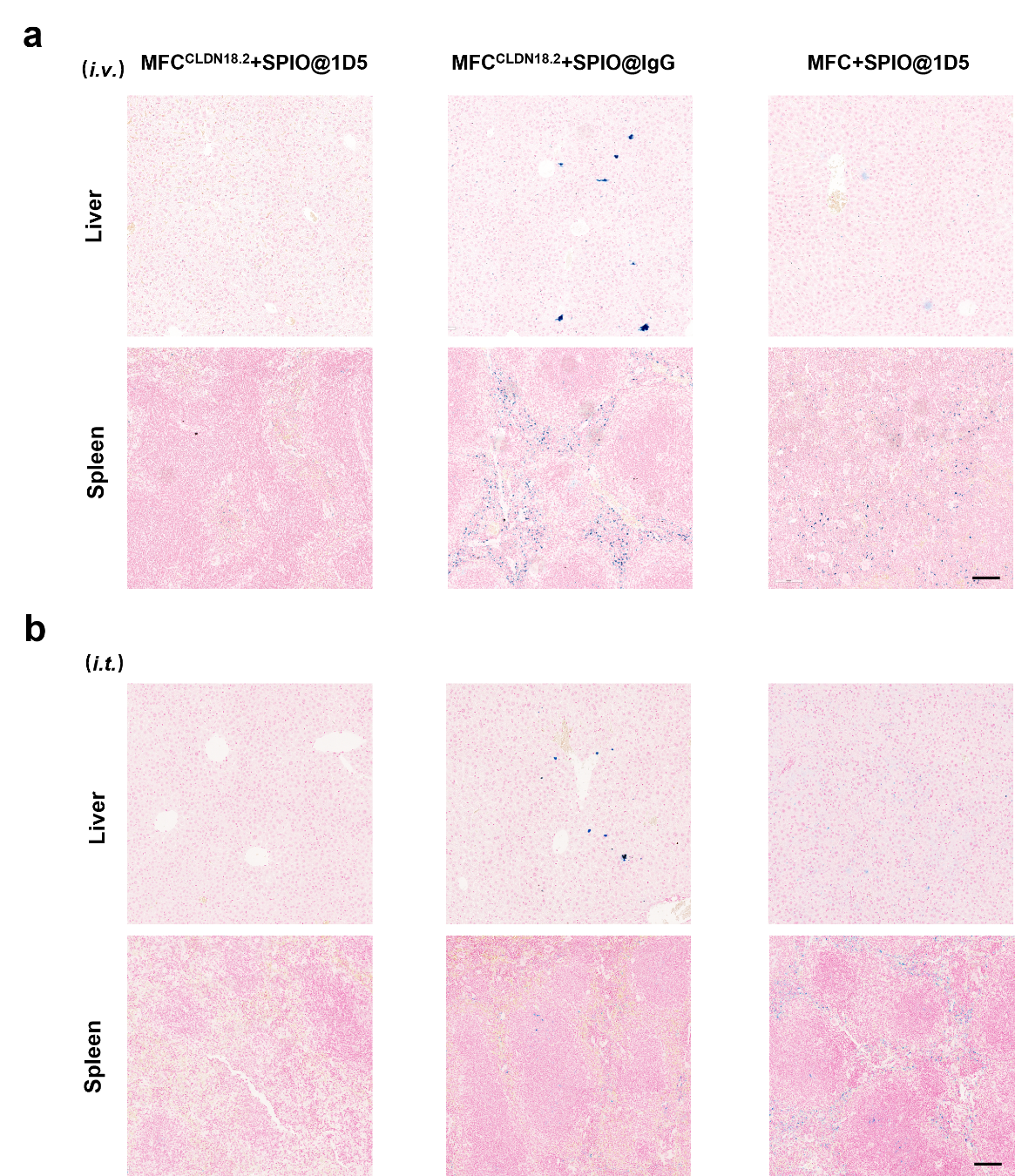


**Figure S19.** Representative Prussian blue staining of the tumor sections after *in vivo* imaging. Prussian blue staining of resected liver and spleen after intravenous (a) and intratumoral (b) injection. Scale bar: 100 μm. *i.v.*: intravenous administration; *i.t.*: intratumoral administration.


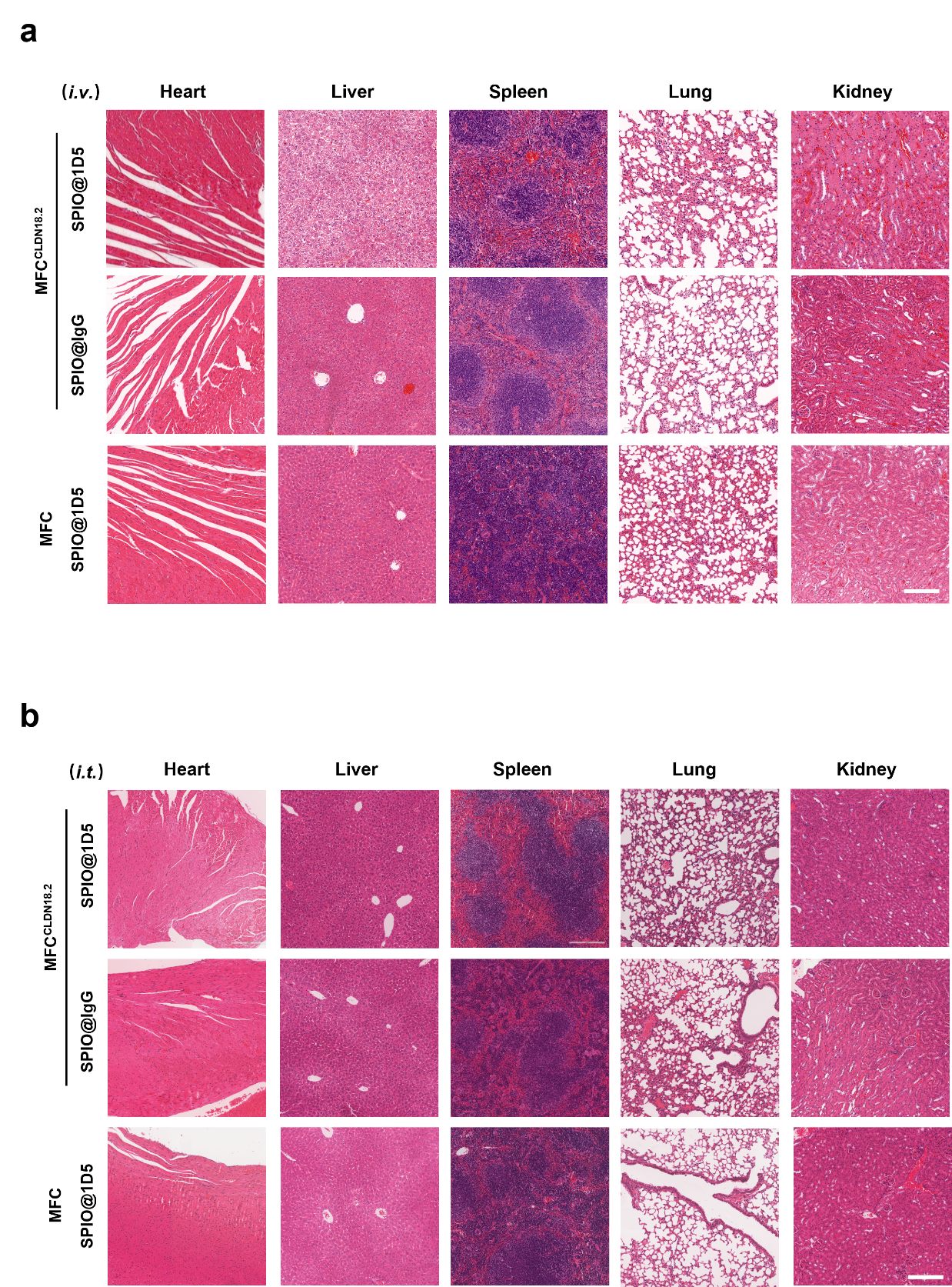


**Figure S20.** Representative histological images for H&E staining were obtained from the heart, liver, spleen, lung, and kidney of MFC^CLDN18.2^-bearing 615 mice with indicated treatments. Tissues were harvested at 48 h post-intratumoral injection. Scale bar: 200 μm. *i.v.*: intravenous administration; *i.t.*: intratumoral administration.


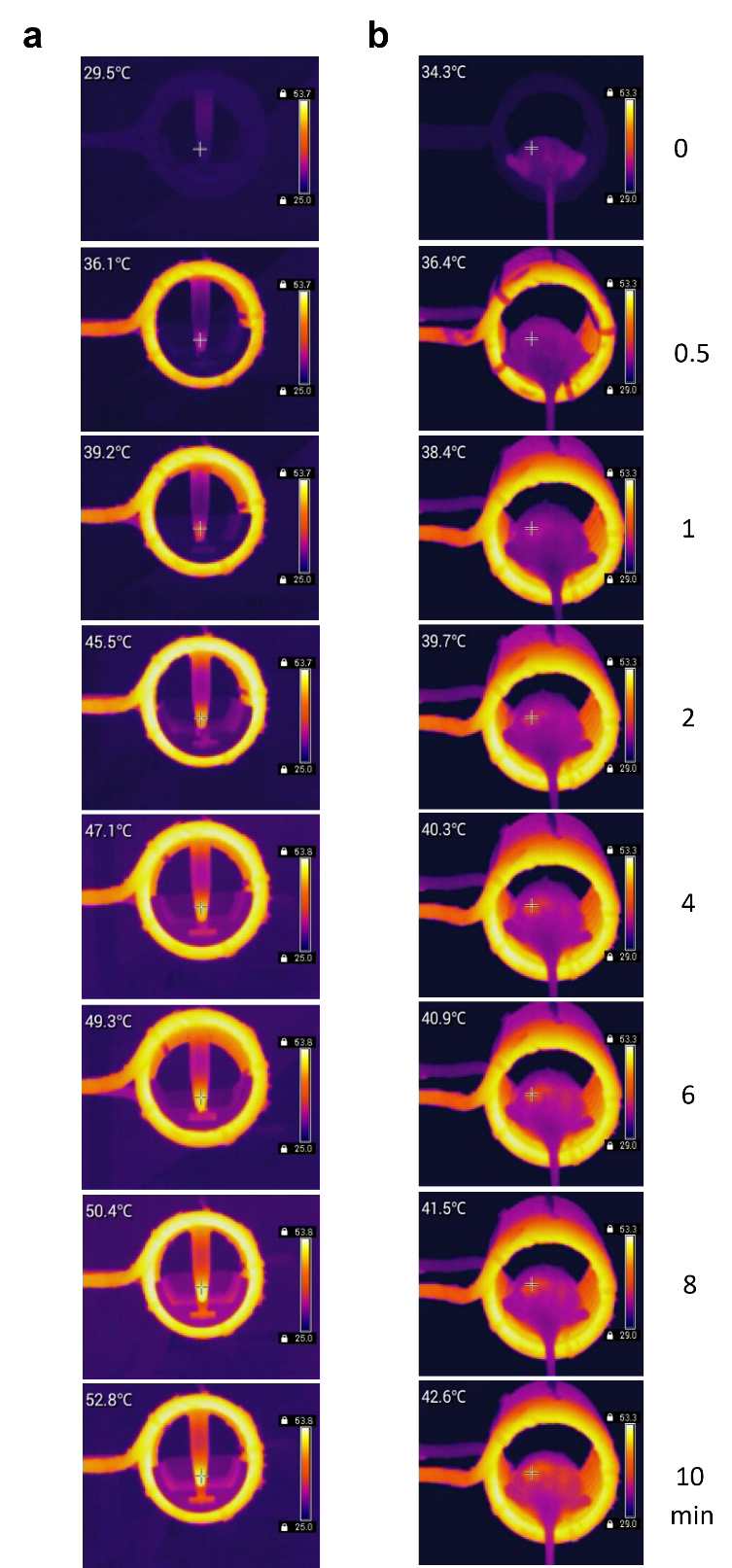


**Figure S21.** Infrared imaging of SPIO@1D5 solution and SPIO@1D5 treated MFC^CLDN18.2^-bearing 615 mice in AMF. a) Infrared imaging of SPIO@1D5 solutions (1.0 mg/mL SPIO) upon AMF treatment (20A, 353KHZ, 1.6KW) for the indicated time intervals (0–10 min). b) Infrared imaging of MFC^CLDN18.2^-bearing 615 mice upon AMF treatment (20A, 353KHZ, 1.6KW) for the indicated time intervals (0–10 min). The mice were treated with SPIO@1D5 by intratumoral administration 12 hours before AMF treatment.


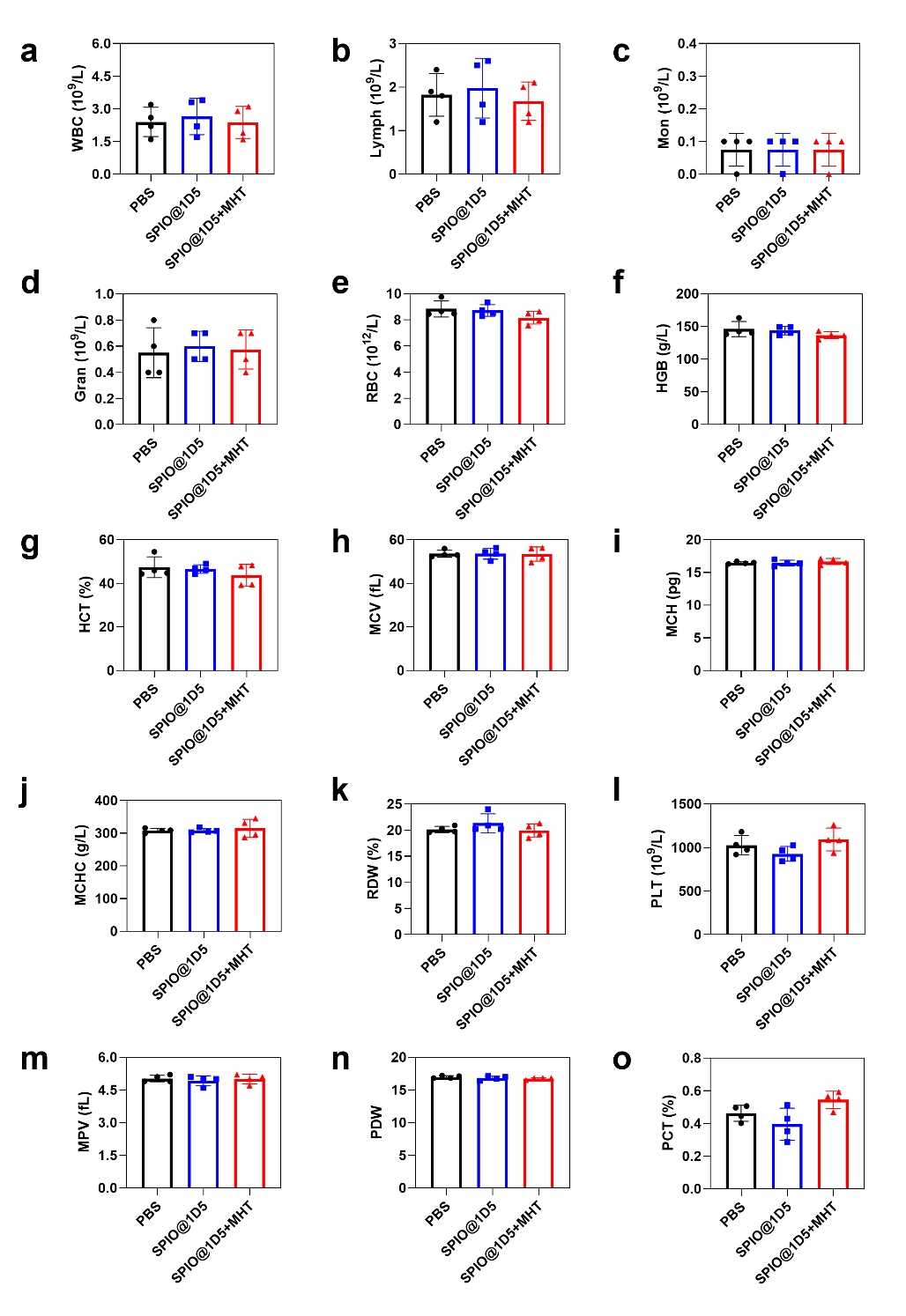


**Figure S22.** Blood routine indexes (white blood cell count (WBC), lymphocyte (Lymph), monocytes (Mon), granulocyte (Gran), red blood cell count (RBC), hemoglobin (HGB), hematocrit (HCT), mean corpuscular volume (MCV), mean corpuscular hemoglobin (MCH), mean corpuscular concentration (MCHC), red blood cell distribution width (RDW), platelets (PLT), mean platelet volume (MPV), platelet distribution width (PDW), and procalcitonin (PCT) of the NOD-scid mice bearing PDX-G1 with the treatment of PBS, SPIO@1D5-ICG or SPIO@1D5-ICG+MHT. Data are expressed as means ± SD (n = 4). Statistical significances were calculated via one-way ANOVA and the Dunnett multiple comparisons test.


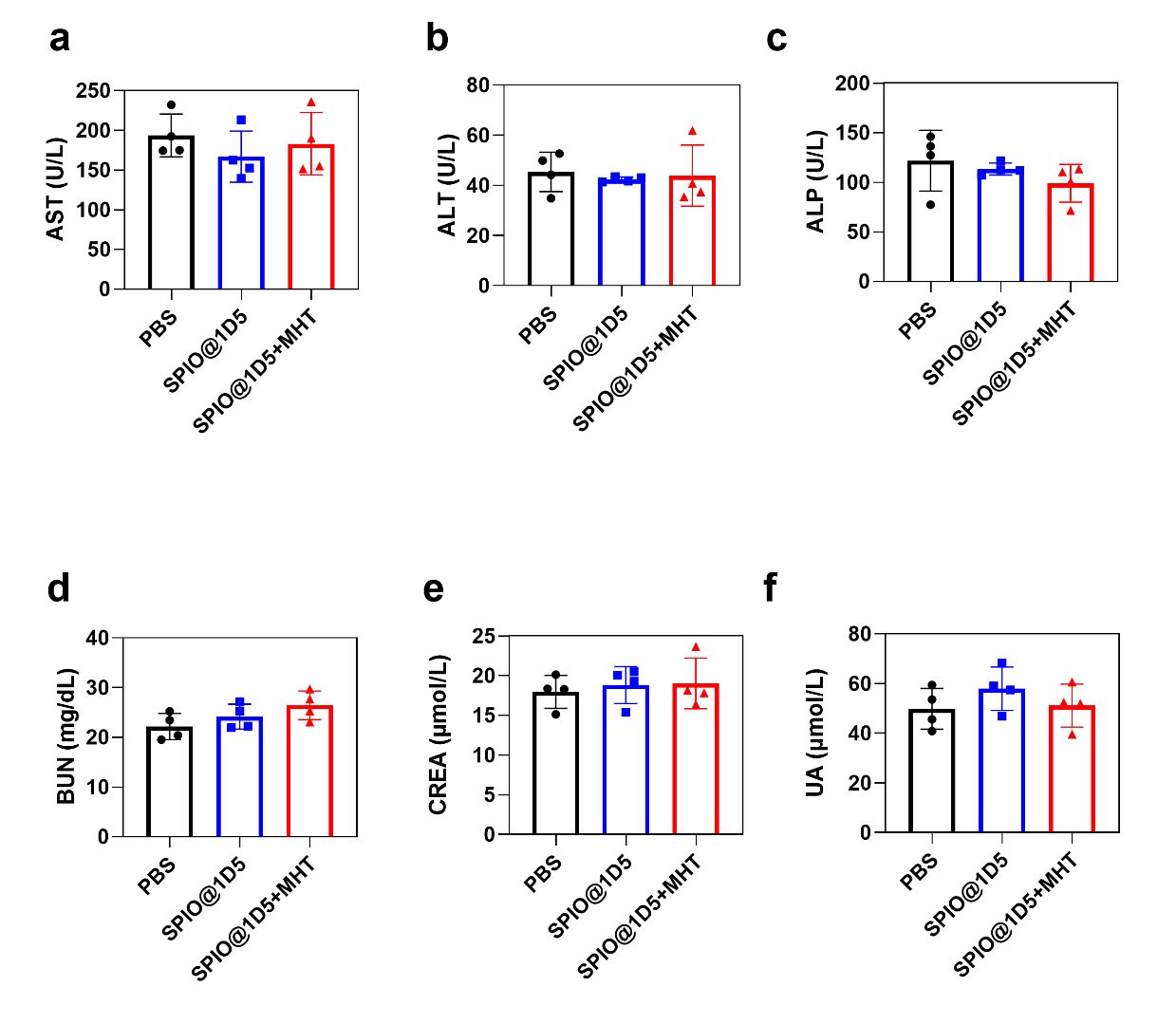


**Figure S23.** Blood test parameters included liver and renal function indexes (alanine transaminase (ALT), aspartate transaminase (AST), alkaline phosphatase (ALP), blood urea nitrogen (BUN), creatinine (CREA), and uric acid (UA)) of the NOD-scid mice bearing PDX-G1 with the treatment of PBS, SPIO@1D5 or SPIO@1D5 +MHT. Data are expressed as means ± SD (n = 4). Statistical significances were calculated via one-way ANOVA and the Dunnett multiple comparisons test.


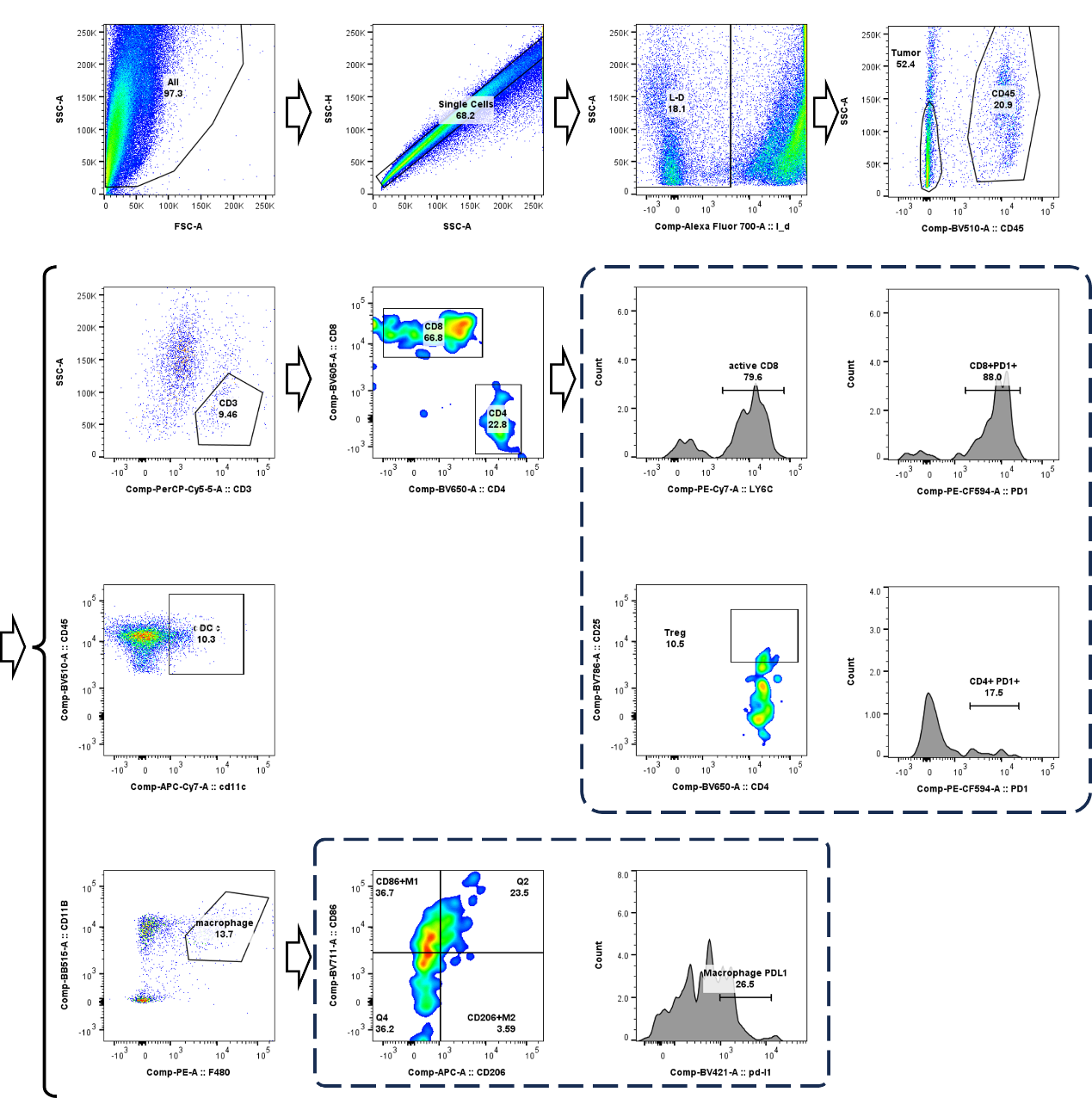


**Figure S24.** Gating strategy for functional lymphocytes (CD8^+^ PD1^+^ cells and CD4^+^ PD1^+^ cells), recruited DC (CD45^+^ CD11c^+^ cells), and M1 and M2 macrophages (F4/80^+^ CD80^+^ cells and F4/80^+^ CD206^+^ cells) in tumours. Related to Figure 6.


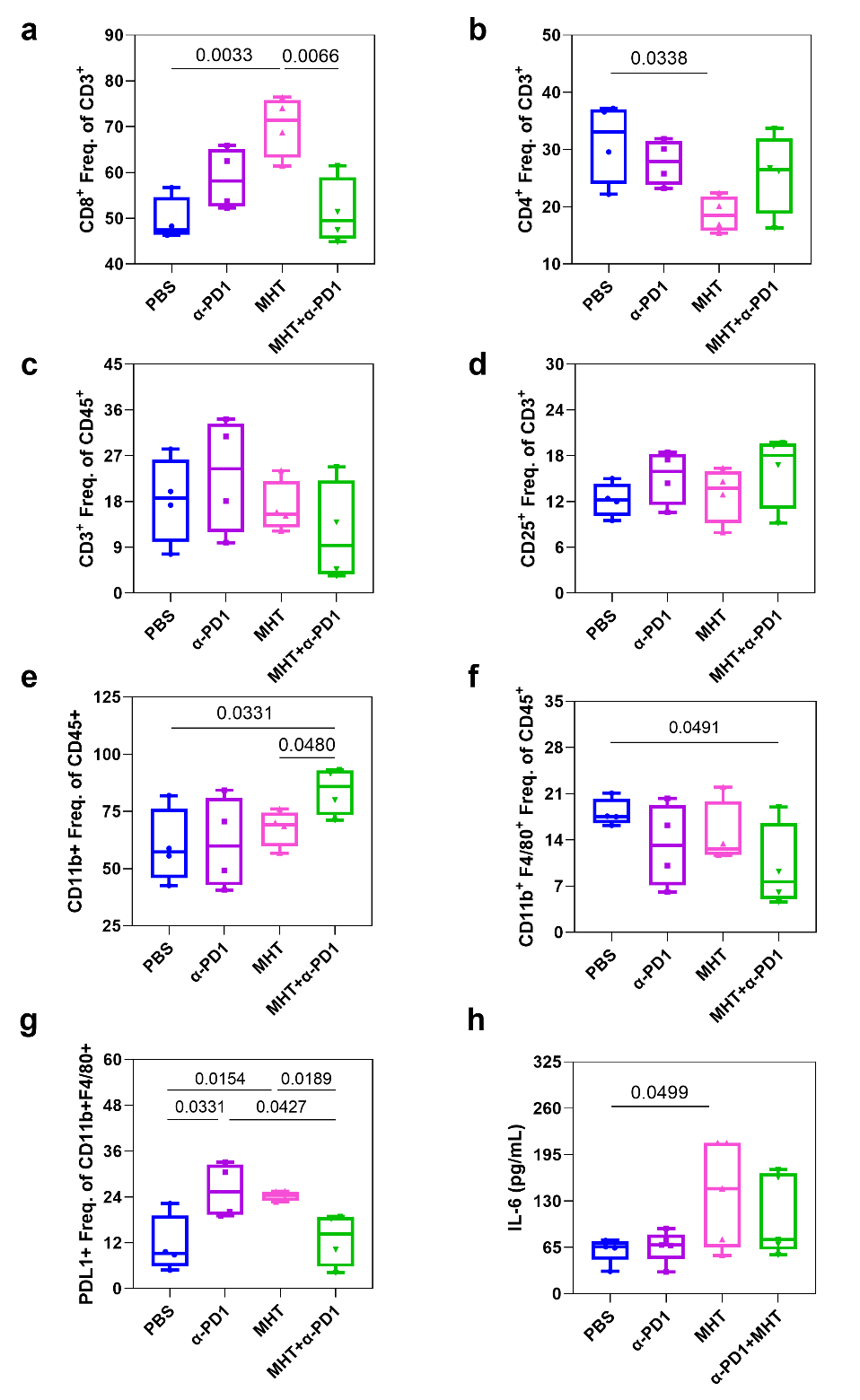


**Figure S25.** Additional quantitative flow cytometry analysis of Fig. 6. a) Percentage of CD8^+^ cells gated on CD3^+^ cells. b) Percentage of CD4^+^ cells gated on CD3^+^ cells. c) Percentage of CD3^+^ cells gated on CD45^+^ cells. d) Percentage of CD25^+^ cells gated on CD3^+^ cells. e) Percentage of CD11b^+^ cells gated on CD45^+^ cells. f) Percentage of CD11b^+^ and F4/80^+^ cells gated on CD45^+^ cells. g) Percentage of PD-L1^+^ cells gated on CD11b^+^ and F4/80^+^ cells. h) Serum levels of IL-6 in the indicated groups. Data are expressed as means ± SD (n = 4). Statistical significances were calculated via one-way ANOVA and Tukey multiple comparisons test.


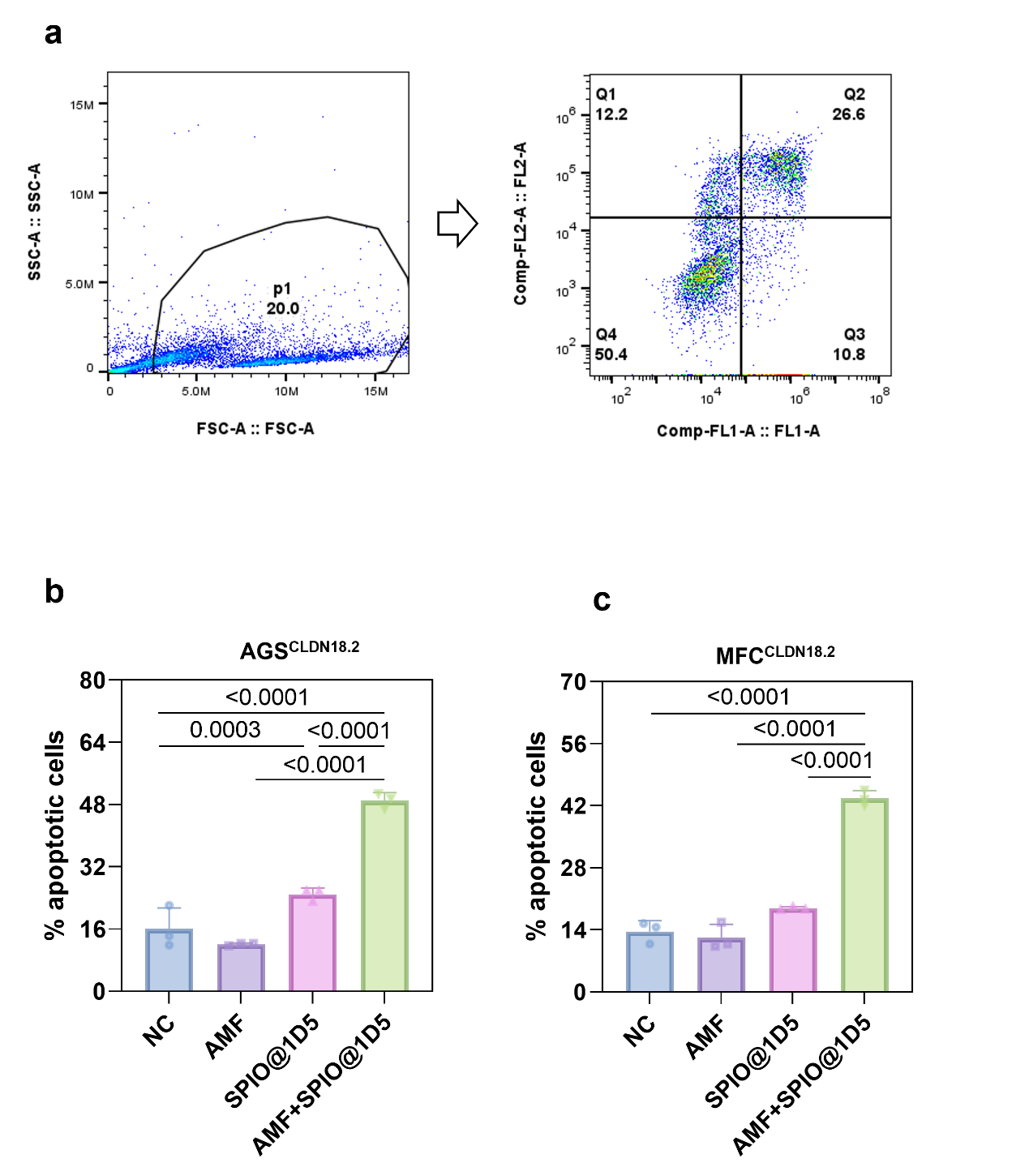


**Figure S26.** Flow cytometry analysis of apoptotic cells after MHT treatments. a) Gating scheme of annexin-V and PI-PE co-stained AGS^CLDN18.2^ and MFC^CLDN18.2^cells. b, c) Quantification of apoptotic cells after indicated treatments in AGS^CLDN18.2^ (b) and MFC^CLDN18.2^ cells (c). Data are expressed as means ± SD (n = 4). Statistical significances were calculated via one-way ANOVA and Tukey multiple comparisons test.


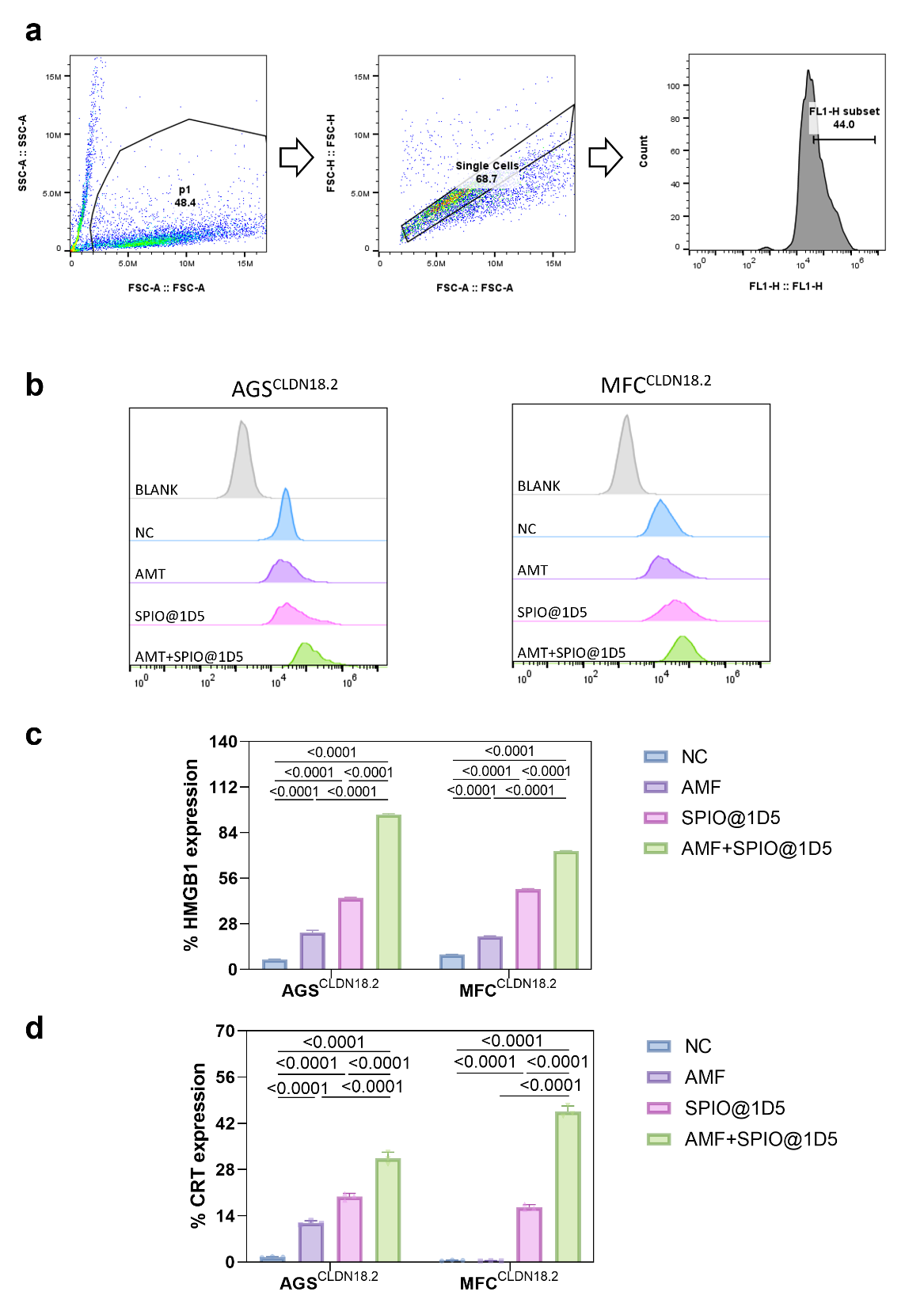


**Figure S27.** Flow cytometry analysis of CRT and HMGB1 in MHT treatments. a) Gating scheme of HMGB1 on AGS^CLDN18.2^ and MFC^CLDN18.2^cells. b) Flow cytometric examination of HMGB1 in AGS^CLDN18.2^ and MFC^CLDN18.2^ cells. c) Quantification of HMGB1 in different indicated groups. d) Quantify CRT in different indicated groups. Data are expressed as means ± SD (n = 3). Statistical significances were calculated via one-way ANOVA and Tukey multiple comparisons test.


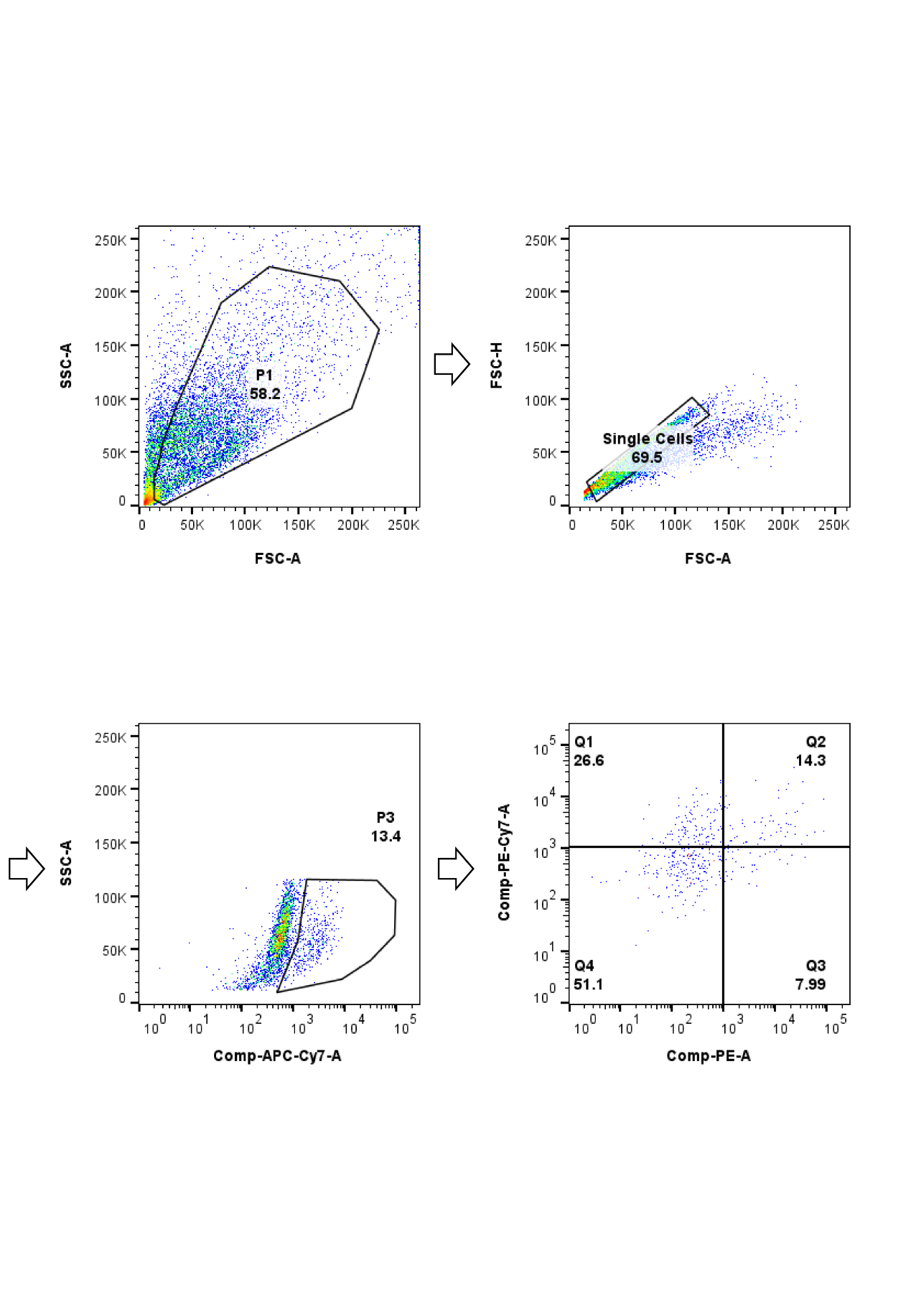


**Figure S28.** The gating scheme for the flow cytometry analysis of the population of BMDCs maturation (CD11c^+^ CD80^+^ CD86^+^) in Figure 7m.


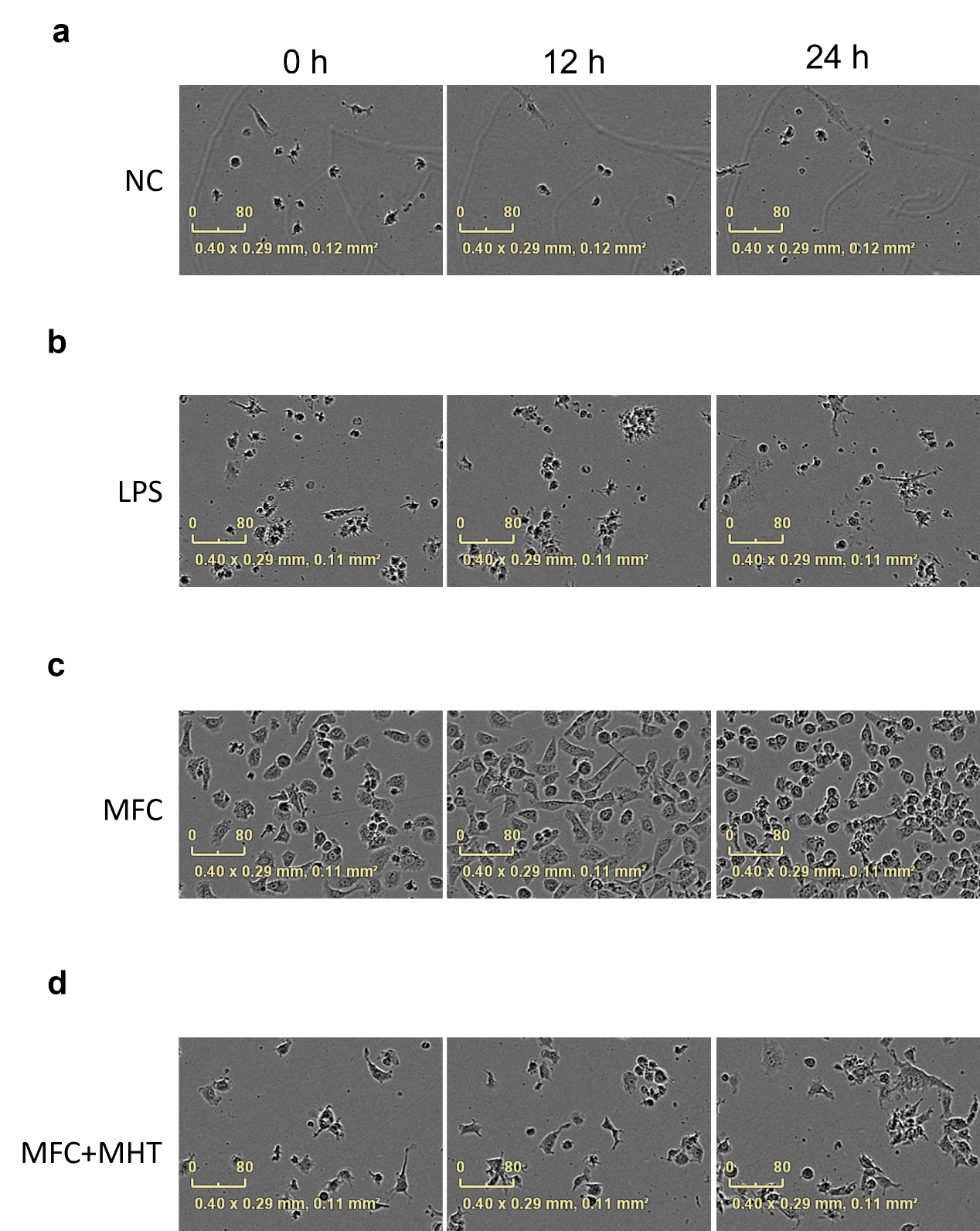


**Figure S29.** Typical morphological changes (IncuCyte Zoom imaging) of BMDC cells after being treated with NC, MHT-treated MFC, MFC, and LPS for 48 h. NC: negative control; LPS: lipopolysaccharide.

**Table S1. Correlation between CLDN18.2 expression levels and clinicopathological features in patients with GC**

| Characteristics | All | CLDN18.2 | | P value |
| --- | --- | --- | --- | --- |
|  |  | Low | High |  |
| Age |  |  |  | **0.030** |
| Median, IQR | 60(52-69) | 60(52-69) | 64.5(54-70) |  |
| Gender |  |  |  | 0.688 |
| Female | 160(28.4%) | 136(28.7%) | 24(26.7%) |  |
| Male | 403(71.6%) | 337(71.3%) | 66(73.3%) |  |
| Location |  |  |  | 0.514 |
| Upper | 5(0.9%) | 4(0.9%) | 1(1.2%) |  |
| Middle | 118(22.1%) | 96(21.3%) | 22(26.2%) |  |
| Lower | 275(51.4%) | 237(52.5%) | 38(45.2%) |  |
| GEJ | 137(25.6%) | 114(25.3%) | 23(27.4%) |  |
| Lauren type |  |  |  | **0.008** |
| Diffused | 136(24.5%) | 123(26.5%) | 13(14.6%) |  |
| Intestinal | 304(54.9%) | 242(52.0%) | 62(69.7%) |  |
| Mixed | 114(20.6%) | 100(21.5%) | 14(15.7%) |  |
| Differentiation |  |  |  | **0.031** |
| Poorly | 248(46.4%) | 216(48.4%) | 32(36.4%) |  |
| Moderately | 268(50.2%) | 218(48.9%) | 50(56.8%) |  |
| Well | 18(3.4%) | 12(2.7%) | 6(6.8%) |  |
| Tumor size |  |  |  | 0.798 |
| <5cm | 291(53.3%) | 243(53.1%) | 48(54.5%) |  |
| ≥5cm | 255(46.7%) | 215(46.9%) | 40(45.5%) |  |
| MVD |  |  |  | **0.003** |
| Median, IQR | 50(35-69) | 51(36-70) | 44(30-58) |  |
| Vascular invasion |  |  |  | 0.599 |
| Negative | 245(44.1%) | 203(43.7%) | 42(46.7%) |  |
| Positive | 310(55.9%) | 262(56.3%) | 48(53.3%) |  |
| Histological type |  |  |  | 0.139 |
| Ade | 490(88.6%) | 407(87.9%) | 83(92.2%) |  |
| MucAde | 11(2.0%) | 8(1.7%) | 3(3.3%) |  |
| Ring | 52(9.4%) | 48(10.4%) | 4(4.4%) |  |
| TNM |  |  |  | **0.025** |
| I-II | 242(49.1%) | 193(46.8%) | 49(60.5%) |  |
| III-IV | 251(50.9%) | 219(53.2%) | 32(39.5%) |  |

Abbreviations: IQR: interquartile range; GEJ: gastroesophageal junction; MVD: microvessel density; Ade: adenocarcinoma; MucAde: mucinous adenocarcinoma.
